# Post-irradiation dietary restriction impairs hematopoiesis via inhibition of the pentose phosphate pathway in hematopoietic stem and progenitor cells

**DOI:** 10.1038/s41419-025-08249-w

**Published:** 2026-01-07

**Authors:** Si Tao, Mingyue Su, Chenghui Yu, Xingxing Qiu, Bing Zou, Rongrong Qiu, Yuanyuan Wu, Lulu Liu, Zhendong Tao, Liu Zhang, Hua Wang, Duozhuang Tang

**Affiliations:** 1 Department of Oncology, The Second Affiliated Hospital, Jiangxi Medical College, Nanchang University, Nanchang, Jiangxi China; 2 Department of Medical Oncology, First Affiliated Hospital, School of Medicine, Zhejiang University, Hangzhou, Zhejiang China; 3 Department of Medical Laboratory Medicine, Jiangxi Province Hospital of Integrated Chinese & Western Medicine, Nanchang, Jiangxi China; 4 Intensive Care Unit, Beijing Jishuitan Hospital, Capital Medical University, Beijing, China; 5 Jiangxi Province Key Laboratory of Precision Cell Therapy (2024SSY06241), The Second Affiliated Hospital, Jiangxi Medical College, Nanchang University, Nanchang, Jiangxi China; 6 Department of Hematology, The Second Affiliated Hospital, Jiangxi Medical College, Nanchang University, Nanchang, Jiangxi China

**Keywords:** Stem-cell research, Experimental models of disease

## Abstract

Although the clinical observation of hematologic toxicity related to radiotherapy has been recognized for a long time, the underlying mechanisms remain to be fully explored. Here, we established a mouse model of reduced dietary intake (dietary restriction, DR, 30% reduction in food intake compared to age-matched and gender-matched mice) following X-ray radiation exposure to investigate the impact of reduced dietary intake on hematopoiesis after irradiation. We found that post-irradiation DR significantly and persistently suppressed hematopoiesis and notably impaired the regenerative capacity of hematopoietic cells. Compared to ad libitum (AL) fed mice, post-irradiation DR led to sustained upregulation of the DNA damage response (DDR) signaling pathway in hematopoietic cells, even 14 days to 1 month after irradiation, along with delayed DNA repair. Further investigation revealed that DR suppressed the post-irradiation activation of the pentose phosphate pathway (PPP). Inhibition of PPP by 6-Aminonicotinamide (6-AN) in AL mice mimicked the impairment of hematopoiesis observed in DR mice, while activation of PPP by AG1 in DR mice rescued the impairment of DNA repair and hematopoiesis in these mice. Additionally, we conducted a retrospective analysis of 101 cancer patients who received pelvic radiotherapy and found that patients with lower Body Mass Index (BMI) experienced more severe reductions in white blood cells (WBCs), neutrophils, and lymphocytes. This study suggests that DR following irradiation inhibits hematopoiesis by suppressing PPP, providing a new approach to addressing radiotherapy-related myelosuppression and potentially offering solutions for improving refractory hematopoietic disorders associated with radiotherapy.

## Introduction

Radiation therapy is one of the main treatments for tumors, with radiation damage to non-tumor tissues being the most common side effect of radiotherapy [1]. The radiation fields of multiple malignant tumors (such as rectal cancer, anal cancer, prostate cancer, bladder cancer, and female reproductive tract tumors) involve hematopoietic tissues like flat bones and long bones. The hematopoietic system is extremely sensitive to radiation [2], with cytopenia being one of the most common complications of radiotherapy [3]. Complications such as recurrent severe leukopenia and increased susceptibility to infection related to radiotherapy, which can even be difficult to recover, may lead to treatment interruption, inability to tolerate subsequent treatments, and severely affect patient survival and prognosis [4].

Hematopoietic stem cell (HSC) function is influenced by multiple factors, including the condition of the HSCs themselves, local niches, and systemic environment [5]. Recent studies showed that dietary restriction (30% reduction in food intake compared to age-matched and gender-matched mice) in non-irradiated (NIR) mice can induce a change in the differentiation tendency of HSCs and significantly inhibit lymphoid hematopoiesis, and continuous DR (dietary restriction) has a strong inhibitory effect on hematopoiesis under steady-state conditions [6, 7]. These studies suggest that reduced dietary intake has an important regulatory role on hematopoiesis under steady-state conditions, but its regulatory effect on irradiated HSCs and hematopoiesis remains to be elucidated. Caloric restriction (CR) has been shown to extend lifespan and slow aging, with its effects varying based on timing, degree, and individual factors [8, 9]. Early-life CR in rodents yields significant lifespan benefits, while adult-onset CR offers more modest effects [9]. Though CR reduces risks of chronic diseases and improves metabolic health, excessive restriction can cause adverse outcomes like anemia and muscle atrophy, especially in lean or elderly individuals [10]. Numerous studies have highlighted the impact of diet on radiation response [11]. Dietary interventions, such as CR [12] and fatty acids [13], increase the radiosensitivity and oxidative DNA damage. Antioxidants (e.g., vitamins C and E, selenium, and polyphenols) have shown potential in mitigating oxidative stress, reducing inflammation, and protecting tissues [11]. Probiotics further contribute by supporting gut health and preventing radiation-induced gastrointestinal injury [14–16]. These findings underscore the critical role of diet in enhancing radiation tolerance and recovery.

Existing literature indicates that exposure to ionizing radiation can also induce significant damage to the bone marrow (BM) environment, which plays a crucial role in maintaining HSCs [17]. Yet whether post-irradiation DR would also have effects on the BM environment remains to be explored [18, 19].

HSCs are the source of hematopoiesis [20, 21]. Previous studies have reported that radiation can activate DNA damage signaling pathways in HSCs, leading to their hematopoietic dysfunction [22–24]. Radiation exposure also activates DNA repair pathways, which require DNA synthesis to varying extents. The pentose phosphate pathway (PPP) is a crucial metabolic route for glucose oxidation, providing ribose-5-phosphate (R5P) for RNA and DNA de novo synthesis, and Nicotinamide adenine dinucleotide phosphate (NADPH) for reductive biosynthesis (e.g., lipid and deoxyribose synthesis) and antioxidant defense. Studies in frog eggs [25] and cell lines [25, 26] have shown that the activation of glucose-6-phosphate dehydrogenase (G6PD, the rate-limiting enzyme of the oxidative branch of the PPP) is associated with increased PPP activity and is necessary for effective DNA repair [25, 27, 28]. However, the role of the PPP in the repair of hematopoietic cell damage following radiation exposure has not been studied.

Overall, HSC regulation after radiation injury involves multiple mechanisms: radiation damages HSCs directly, and also impairs the BM environment, which supports HSC regeneration [5, 22]. Despite these known mechanisms, the role of DR and the PPP in regulating hematopoiesis post-radiation remains unclear.

Tumor patients often experience reduced appetite and decreased dietary intake due to disease, treatment, and psychological factors, leading to weight loss and a decrease in body mass index (BMI) [29]. It is common for patients undergoing radiotherapy to simultaneously receive chemotherapy, which can further exacerbate appetite reduction and decreased dietary intake. Many studies have shown that clinically, patients receiving radiotherapy commonly experience reduced dietary intake [30]. Some studies suggest that obesity may increase the incidence and overall mortality in patients with major diseases such as cancer [31] or heart failure [32], while other studies indicate that obesity can have a protective effect on patient survival in certain cases [33–35]. These findings have sparked discussions about the obesity paradox, suggesting that obesity may offer protective benefits under specific conditions. It remains unclear whether dietary intake and nutritional status play a role in the regulation of hematopoiesis after radiation damage.

To investigate the impact of reduced dietary intake on hematopoiesis after irradiation, we first established a mouse model of reduced dietary intake (dietary restriction, DR) in C57BL/6 J mice following X-ray radiation exposure. We found that post-irradiation DR significantly and persistently suppressed hematopoiesis and notably impaired the regeneration capacity of HSCs. Comparing to ad libitum (AL) fed mice, post-irradiation DR in these mice led to sustained upregulation of the DNA damage response (DDR) signaling pathway in hematopoietic cells, even 14 days to 1 month after irradiation, along with delayed DNA repair. Further investigation revealed that DR inhibits DNA repair and reduces the anti-reactive oxygen species (ROS) response by suppressing the post-irradiation activation of the PPP, thereby inhibiting hematopoiesis. We also conducted a retrospective analysis of the relationship between blood routine test results and BMI in 101 cancer patients who received pelvic radiotherapy. We found that patients with lower BMI had more severe reductions in white blood cells (WBCs), neutrophils, and lymphocytes. Therefore, we hypothesize that post-irradiation food restriction suppresses PPP in HSCs, thereby reducing the supply of precursors for DNA synthesis and inhibiting DNA repair. This study suggests that DR following irradiation inhibits hematopoiesis by suppressing the PPP, providing a new approach to addressing radiotherapy-related myelosuppression and potentially offering solutions for improving refractory hematopoietic disorders associated with radiotherapy.

## Methods and material

### Mice and dietary intervention

Female C57BL/6J mice aged 2 months were maintained on a 12-h light/dark cycle under specific pathogen-free conditions. Mice were housed individually so that the daily food consumption of each mouse could be determined. This was measured every day for one week to determine their AL-feeding rate. After the 1-week measurement, the average amount of food was determined for every mouse. Animals were randomized into different groups (*n* = 5 mice per group from 1 experiment representative of 2 independent experiments). During the feeding protocol, the ad libitum (AL) mice were fed with unlimited access to food, while DR mice were fed with 70% the average amount of food according to the previous calculation. The calculated 70% food pellet was added to each cage daily at the same time, and was constant over the whole DR period. The mice were fed a commercially formulated diet specifically designed for the growth and reproduction of rats and mice (Tianjin Ke’shi Feed Co., Ltd., Product No. 101670654803973120). The diet ingredients included corn, soybean meal, fish meal, wheat flour, yeast powder, vegetable oil, salt, and various vitamins and mineral elements. The guaranteed nutritional composition was as follows: moisture ≤ 10%, crude fat ≥ 4%, crude ash ≤ 8%, crude protein ≥ 20%, crude fiber ≤ 5%, calcium 1.0–1.8%, and phosphorus 0.6–1.2%. Detailed nutritional analysis values (per kilogram content) are provided in Supplementary Table 1. Animal experiment has been reviewed and approved by the Animal Ethics Committee of Nanchang Leyou Biotechnology Co., Ltd., with the approval number RYE2022062801. Sample size was chosen empirically based on our previous experience in the calculation of experimental variability. The investigators were not blinded to allocation during the experiments and the evaluation of the results.

### Mouse irradiation

A commercial medical electronic linear accelerator (Elekta VersaHD) was used for the radiation experiments. The position of irradiated samples was set at source-to-surface distance 50 cm from the source of the machine. Around 35×35 cm² was set for the radiation field size. The beam used was 6 MV X-ray with a dose rate of 600 MU/min. The daily dose output was checked using a commercial Farmer ion chamber PTW 30013, which was calibrated by a secondary standard dosimetry laboratory. The prescribed doses used in this research were 4.5 and 9 Gy, respectively.

### Flow cytometry

Mouse BM cells were obtained by crushing all hind limbs in sterile Phosphate Buffered Saline (PBS) supplemented with 2% heat-inactivated fetal bovine serum and filtering through a 40 μm cell strainer. BM cells were then resuspended in red blood cell (RBC) lysis buffer to remove RBCs, washed with sterile PBS, manually counted using a hemocytometer, and incubated with antibody mixtures for flow cytometric analysis to detect cell surface markers. For CMPs/GMPs/HSCs, the following antibodies were used: biotinylated anti-B220 (BioLegend, Clone: RA3-6B2, Cat. #103204), CD11b (BioLegend, Clone: M1/70, Cat. #101204), Gr-1 (BioLegend, Clone: RB6-8C5, Cat. #108404), TER-119 (BioLegend, Clone: TER-119, Cat. #116204), CD4 (BioLegend, Clone: RM4-5, Cat. #100508), CD8 (BioLegend, Clone: 53-6.7, Cat. #100704), PE-Cy7-CD16/32 (BioLegend,. Clone: 93, Cat. #101318), FITC-CD34 (BD, Clone: RAM34, Cat. #553733), APC-c-Kit (BioLegend, Clone: 2B8, Cat. #105812), PE-Sca-1 (BioLegend, Clone: D7, Cat. #108108), APC-Cy7-Streptavidin (BioLegend, Cat. #405208), PerCP/Cy5.5-CD150 (BioLegend, Clone: TC15-12F12.2, Cat. #115922). HSCs were defined as Lineage^−^; c-Kit^+^; Sca-1^+^; CD34^−^ and CD150^+^. CMPs were defined as Lineage^−^; c-Kit^+^; Sca-1^−^; CD34^+^ and CD16/32^−^. GMPs were defined as Lineage^−^; c-Kit^+^; Sca-1^−^; CD34^+^ and CD16/32^+^. MEPs were defined as lineage^−^; c-Kit^+^Sca-1^−^; CD34^−^; and CD16/32^−^ cells. B220 cells in BM, peripheral blood, and spleen were stained with the APC-Cy7-B220 (BioLegend, Clone: RA3-6B2, Cat. #103224) antibody. Bone marrow resident macrophages (BMRMs) were stained with the FITC-CD169 (BioLegend, Clone: 3D6.112, Cat. #142406), PerCP/Cy5.5-CD115 (BioLegend, Clone: AFS98, Cat. #135526), PE-F4/80 (BioLegend, Clone: BM8, Cat. #123110), APC-Gr-1 (BioLegend, Clone: RB6-8C5, Cat. #108412) antibodies. BMRMs were defined as: Gr-1^med/−^, CD115^−^, F4/80^+^, SSC^−^, CD169^+^. For apoptosis analysis, the staining method for HSC surface markers included a mixture of biotinylated anti-B220 (Clone: RA3-6B2, Cat. #103204), CD11b (Clone: M1/70, Cat. #101204), Gr-1 (Clone: RB6-8C5, Cat. #108404), TER-119 (Clone: TER-119, Cat. #116204), CD4 (Clone: GK1.5, Cat. #100404), and CD8 (Clone: 53-6.7, Cat. #100704) antibodies (BioLegend), along with FITC-CD34 (BD, Clone: RAM34, Cat. #553733), APC-c-Kit (BioLegend, Clone: 2B8, Cat. #105812), PE-Cy7-Sca-1 (BioLegend, Clone: D7, Cat. #558162), APC-Cy7-Streptavidin (BioLegend, Cat. #405208), and PerCP/Cy5.5-CD150 (BioLegend, Clone: TC15-12F12.2, Cat. #115922). After staining, cells were fixed using paraformaldehyde (Servicebio, Cat. #G1101-500ml) and subsequently stained according to the manufacturer’s instructions with the BD Pharmingen™ PE Annexin V Apoptosis Detection Kit I (BD, Cat. #559763). Cells were incubated in PE-Annexin V (Cat. #51-65875X) at room temperature in the dark for 15 min, followed by DAPI (Absin, Cat. #abs42016321) binding buffer staining to detect DNA content. Stained cells were analyzed on a flow cytometer (FACS) (FACS Canto II; BD).

For ROS staining, CellROX™ Green (Thermo Fisher Scientific, cat. #C10492) was used to treat 1 × 10^6^ total BM cells to measure intracellular ROS levels. CellROX™ Green is a fluorescent probe that emits bright green, photostable fluorescence upon oxidation. For the negative control, 500 µM N-acetylcysteine was added and incubated at 37 °C and 5% CO_2_ for 1 h. Then, 200 µM tert-butyl hydroperoxide was added to both the positive and negative controls and incubated under the same conditions for 30 min. Next, 250 µM CellROX™ Green reagent was added to 1 ml of the cell suspension and incubated at room temperature, protected from light, for 30 min. Finally, the green fluorophore was excited by a 488 nm laser, and FACS (FACS Canto II; BD) was used for analysis. The gating was adjusted using the positive and negative controls, MIF = 1 in NIR AL mice.

### Sorting

For hematopoietic stem and progenitor cells (HSPCs) sorting, BM cells were labeled with APC anti-mouse CD117 (c-Kit) antibody (BioLegend, Clone: 2B8, Cat. #105812), incubated at 4 °C for 30 min in the dark, and then enriched and sorted using anti-APC MicroBeads (Miltenyi Biotec, Cat. #130-090-855) and LS Columns (Miltenyi Biotec, Catalog no. 130-122-729) following the manufacturer’s instructions.

### 6-aminonicotinamide treatment

The 6-aminonicotinamide (6-AN) was purchased from MedChemExpress Co., Ltd, China (CAS No. 329-89-5; Lot number: HY-W010342; purity: 99.95%), and was diluted in saline for injection intraperitoneally for 7 days post-irradiation. The daily dose of 6-AN was 10 mg/kg for young mice. In the control group, saline was injected instead of 6-AN.

### G6PD activator AG1 treatment

The G6PD activator AG1 was purchased from MedChemExpress Co., Ltd., China (CAS No. 421581-52-4; Lot number: HY-123962; purity: 99.54%), and was diluted in dimethyl sulfoxide (DMSO) for injection intraperitoneally every other day for 9 times post-irradiation. The daily dose of AG1 was 20 mg/kg for young mice. The control group was injected with an equal volume of DMSO.

### Sucrose water use

Sucrose was purchased from Servicebio Co., Ltd, China (CAS No. 57-50-1; Lot number: GC205014-500g; purity: analytical reagent). The sucrose drinking solution was prepared at a concentration of 50 g/L according to published protocols, with the solution being replaced every 2–3 days.

### G6PD activity determination

G6PDH activity was measured using a commercial kit (#S0189, Beyotime, Shanghai, China) according to the manufacturer’s instructions. Briefly, 1 × 10^6^ total BM cells were treated with 200 μL extraction buffer, centrifuged at 12,000 × *g* for 10 min, and 50 μL of supernatant was incubated with 50 μL of G6PDH working solution in the dark at room temperature for 10 min. Absorbance was measured at 450 nm.

### NADPH content determination

NADPH content was assessed using a NADPH detection kit (#S0179, Beyotime, Shanghai, China) according to the manufacturer’s instructions. Briefly, 1 × 10^6^ total BM cells were treated with 200 μL extraction buffer, centrifuged at 10,000 × *g* for 10 min, and the supernatant was incubated at 60 °C for 30 min to decompose NADP. After cooling on ice, the supernatant was reacted with the working solution at 37 °C for 20 min, and absorbance was measured at 450 nm.

### Transplantation

For the primary BM transplantation, 2 × 10^6^ BM cells from irradiated Ly5.2 donor mice were mixed with 1 × 10^6^ BM cells from Ly5.1/5.2 heterozygous competitor mice and injected into lethally irradiated (9 Gy) Ly5.1 recipient mice for competitive transplantation. Four months post-primary transplantation, 10 × 10^6^ BM cells from the primary recipients were serially transplanted into lethally irradiated (9 Gy) Ly5.1 recipient mice. Peripheral blood (PB) analyses of the primary and secondary transplantations were performed at 1–4 months after transplantation. The Ly5.1/5.2 competitor mice correspond to NIR AL mice.

### Immunofluorescence staining

C-Kit^+^ cells were resuspended to 1 × 10² cells/μL, dropped onto positively charged slides (Thermo, Catalog no. 4951PLUS), fixed with 4% paraformaldehyde (Servicebio, Catalog no. G1101) at room temperature (RT) for 10 min, permeabilized with 0.25% Triton/PBS at RT for 10 min, and blocked with 1% Bovine Serum Albumin (BSA)/PBS at RT for 1 h. Cells were then incubated overnight at 4 °C with anti-phosphorylated histone H2AX antibody (Abcam, Catalog no. Ser139, Cat. #ab22551) and Anti-53BP1 antibodies (Abcam, Cat. #ab175933) at 1:200 dilution, followed by incubation with anti-mouse Alexa Fluor 488 secondary antibody (Abcam, Cat. #ab237174) and anti-rabbit Alexa Fluor 594 secondary antibody (Abcam, Cat. #ab150080) at 1:400 dilution at RT for 1 h. Nuclei were counterstained with DAPI. Images were acquired on a Leica SP5 fluorescence microscope and processed with LAS-AF-Lite_2.6.0. Foci in 250 c-Kit^+^ cells per group were manually counted and analyzed.

BM cells were washed with ice PBS twice, and then fixed with 4% paraformaldehyde (Servicebio, Cat. no. G1101) at room temperature (RT) for 10 min, permeabilized with 0.25% Triton/PBS at RT for 10 min, and blocked with 1% BSA/PBS at RT for 1 h. After blocking, cells were incubated with Nrf2 Polyclonal Antibody (1:200, Invitrogen, Thermofisher, Cat. #PA5-27882) overnight at 4 °C. After washing, cells were incubated for 1 h with Alexa Fluor 488 (1:400, Invitrogen, Thermofisher, Cat. #A31731) secondary antibody at 37 °C and stained with DAPI to label cell nuclei. The signals were visualized, and digital images were obtained by the fluorescence stereoscope (Olympus SZX16). Foci in 250 BM cells per group were manually counted and analyzed.

### Comet assay

Sorted c-Kit^+^ cells were resuspended in PBS at 1 × 10^6^ cells/ml, mixed with 0.5% low-melting-point agarose (Beyotime, Cat. no. 39346-81-1), and quickly spread onto slides pre-coated with 0.8% normal-melting-point agarose (Thermo, Cat. no. 16500100). Slides were solidified at 4 °C for about 5 min, lysed under alkaline conditions for 1–2 h, and electrophoresed at 4 °C with buffer pH > 13. After electrophoresis, DNA was stained with propidium iodide (MCE, Cat. no. HY-D0815). DNA damage was observed under a fluorescence microscope (Nikon), and tail DNA percentage was analyzed using CASP software.

### HE staining

Femurs were collected and fixed in 4% paraformaldehyde (Servicebio, Catalog no. G1101) at RT for 24 h, decalcified in 0.5 M EDTA (pH = 7.4, Servicebio, China, G1105) for 2 weeks, and then embedded in paraffin. Sections were cut at a thickness of 5 μm. Paraffin sections were deparaffinized in xylene twice for 20 min, rehydrated in gradient alcohol, stained with hematoxylin (Servicebio, Catalog no. G1001) for 5 min, washed with tap water, counterstained with eosin (Servicebio, Catalog no. G1004) for 5 min, dehydrated in gradient alcohol, and cleared in xylene. Sections were mounted with neutral gum and examined under a light microscope (Nikon).

### Oil red O staining

Oil red O staining was performed according to BioVision’s manual (BioVision). Briefly, femur frozen sections were equilibrated to RT, washed with 60% isopropanol (MCE, Catalog no. 67-63-0), stained with oil red O solution (Sigma-Aldrich, Cat. no. O1391) at 37 °C for 15 min, counterstained with hematoxylin (Servicebio, Catalog no. G1001) for 10 min, mounted with glycerol gelatin, and examined under a light microscope (Nikon).

### Total RNA extraction and reverse transcription

Total RNA from cell samples was extracted using RNAsimple Total RNA Kit (TianGen Biotech) and reverse-transcribed using RevertAid First Strand cDNA Synthesis Kit (Thermo Scientific) according to the manufacturer’s instructions. The process included incubation at 42 °C for 60 min and inactivation at 70 °C for 5 min.

### Real-time quantitative PCR (qPCR)

qRT-PCR was performed using TransStart Tip Green qPCR SuperMix (TransGen Biotech) on an ABI 7900 real-time PCR system (Applied Biosystems) in triplicate. Reaction conditions were: 94 °C for 30 s, followed by 40 cycles of 94 °C for 30 s and 60 °C for 30 s. Relative gene expression was normalized to β-actin in each sample, with the NIR AL group normalized to 1. Primer sequences used were listed in Supplementary Table 2.

### Peripheral blood cell counting

PB was collected from the orbital venous plexus into tubes containing 0.5 M EDTA. PB cell counts were assessed using an automated blood analyzer (Sysmex, XS-500i).

### Clinical data collection

Clinical data were retrospectively collected from 101 patients receiving radiotherapy at the Second Affiliated Hospital of Nanchang University. Data collection began on the first day of radiotherapy, and the BMI values, as well as the absolute counts of WBCs, lymphocytes, monocytes, RBCs, and platelets, were recorded weekly for the patients over a period of one month. The groups with differences were used to plot the receiver operating characteristic curve (ROC) and calculate the area under the curve (AUC) value to evaluate the model quality and select the optimal model threshold. The use of human data in this study was approved by the Institutional Ethics Committee of the Second Affiliated Hospital of Nanchang University (Approval No. MR-36-25-024749). Informed consent was waived by the Institutional Ethics Committee of the Second Affiliated Hospital of Nanchang University because of the retrospective nature of this study. All procedures involving human data adhered to the Declaration of Helsinki (2013 revision). As this was a retrospective study, the requirement for informed consent was waived by the Institutional Ethics Committee. Baseline demographics and disease characteristics of patients (*n* = 101) are presented in Supplementary Table 3.

### Radiotherapy and pelvic bone marrow delineation

The radiotherapy was performed using either intensity modulated radiotherapy (IMRT) or volumetric modulated radiotherapy (VMAT) with an Elekta linear accelerator (Elekta Versa HD, Sweden). The pelvic BM and other organs at risk (OARs) were contoured by a commercial segmentation software that worked with an artificial intelligence algorithm. Contouring of the targets (GTV, CTV) was performed by the same senior physician, and the treatment plans were designed by the same experienced dosimetrist. The mean dose (Dmean) of pelvic BM was obtained from the dose-volume histogram (DVH) curves calculated by the treatment planning system Monaco (Elekta, Sweden).

### Statistical analysis

All data were analyzed using GraphPad Prism 9.0 software. Two-group datasets were analyzed using Student’s *t*-tests. For comparison among different groups, one-way or two-way ANOVA was used, with Tukey’s multiple comparisons test was used to calculate *p*-values. Specific data analyses were described in each figure legend. The variance was similar between the groups that were being statistically compared. The results are presented as the mean ± SD. All in vitro experiments were conducted in two independent experiments. *p* < 0.05 was considered statistically significant. Statistical significance was denoted as *p < 0.05, **p < 0.01,***p < 0.001, ****p < 0.0001 and “ns”indicates no significant difference. The number of animals used in each experiment is indicated in the corresponding figure legend. The samples in this study represent biological replicates. Each replicate was independently obtained from different mice under identical experimental conditions. Specifically, BM cells were collected from five mice per group, with each mouse considered as one biological replicate.

## Results

### Post-irradiation DR significantly inhibits hematopoieisis

**Fig. 1:**
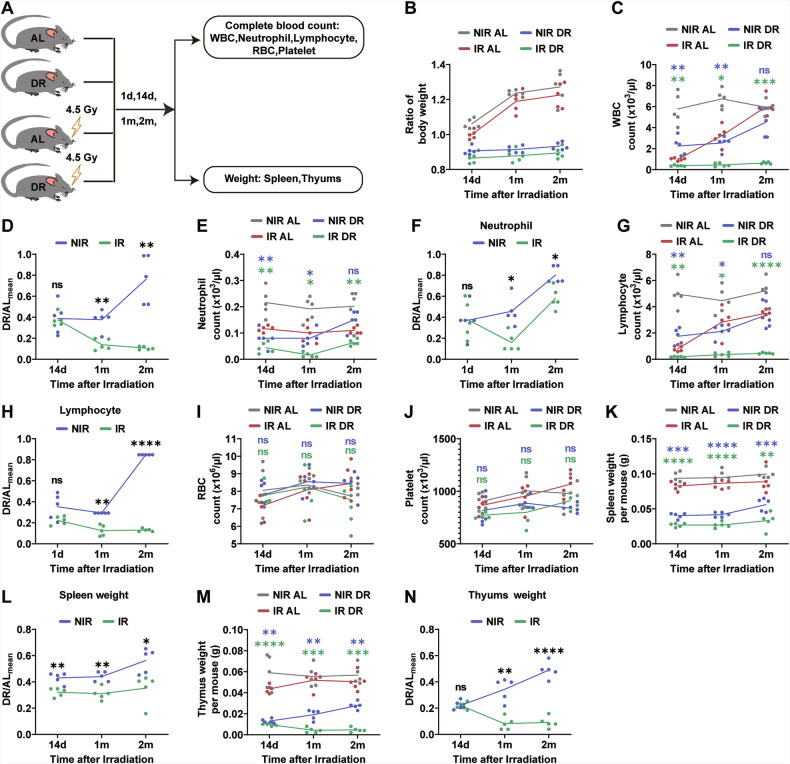
Suppression of body weight and peripheral blood in mice by DR post-irradiation. **A** Experimental scheme. Wild-type C57BL/6 mice (2 months old) were irradiated with 4.5 Gy X-rays, fed with either AL diet or DR diet (daily food intake restricted to 70% of the intake of age- and sex-matched AL mice). NIR mice receiving an AL or DR diet were also monitored as a control (*n* = 5 mice per group from 1 experiment representative of 2 independent experiments). **B** Ratio of body weight of mice at the indicated time points to the average initial body weight post-irradiation and no irradiation. **C**–**J** PB cell counting at the indicated time points. Absolute counting value of WBC (**C**), Nitrification (**E**), Lymphocytes (**G**), RBC (**I**) and Platelet (**J**). **D**, **F**, **H** Ratio of the number of WBC (**D**), Neutrophil (**F**), Lymphocytes (**H**) in DR mice at each time point to the average number in AL mice. Spleen (**K**) and thymus weight (**M**) at the indicated time points. Ratio of spleen weight (**L**) and thymus weight (**N**) in DR mice at each time point to the average spleen or thymus weight in AL mice. Results were displayed as mean ± SD; ns, not significant; *p < 0.05; **p < 0.01; ***p < 0.001; ****p < 0.0001 by Two-way ANOVA test and multiple comparisons. AL ad libitum feeding, DR dietary restriction, NIR AL no irradiated ad libitum group, NIR DR no irradiated dietary restriction group, IR AL irradiated ad libitum group, IR DR irradiated dietary restriction group, WBC white blood cell, RBC red blood cell, d days, m months. The purple symbols indicate statistical significance between the non-irradiated groups. The green symbols indicate statistical significance between the irradiated groups.

**Fig. 2:**
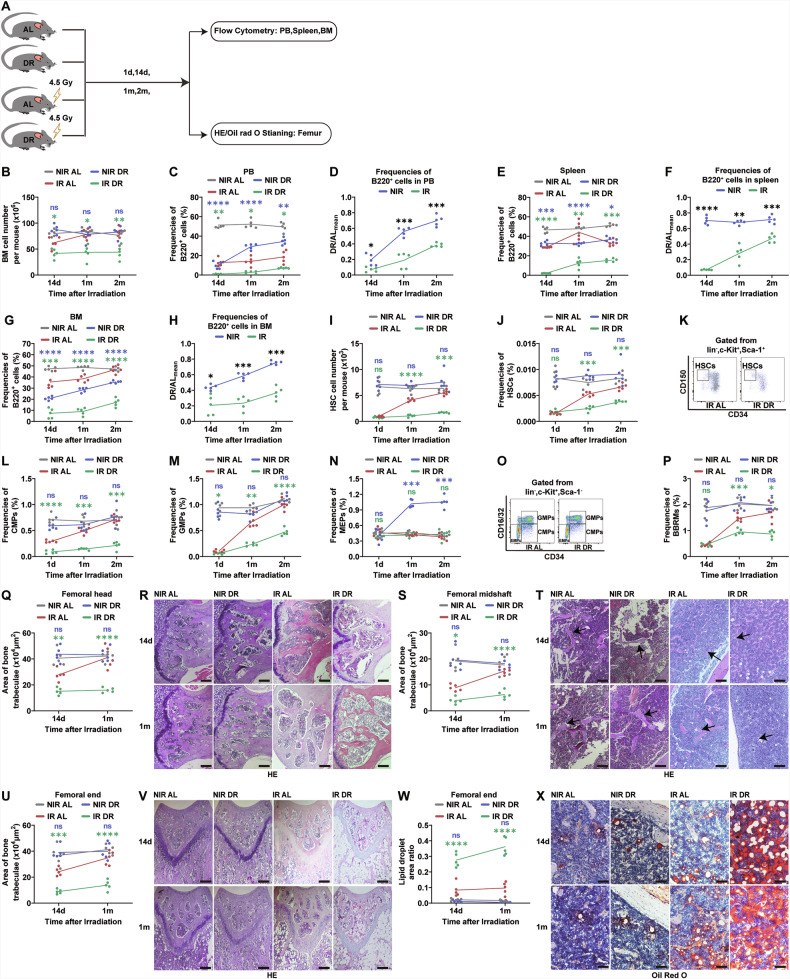
Suppression of hematopoiesis and damage to hematopoietic microenvironment in mice by DR post-irradiation. **A** Experimental scheme. Wild-type C57BL/6 mice (2 months old) were irradiated with 4.5 Gy X-rays, fed with either AL diet or DR diet (daily food intake restricted to 70% of the intake of age- and sex-matched AL mice). NIR mice receiving AL or DR diet were also monitored as a control (*n* = 5 mice per group from 1 experiment representative of 2 independent experiments). **B** Total BM cells counts at indicated time points post-irradiation and no irradiation. FACS analysis of frequencies of B220^+^ lymphocytes in PB (**C**, **D**), spleen (**E**, **F**), BM (**G**, **H**); and frequencies of HSCs (**J**), CMPs (**L**), GMPs (**M**), MEPs (**N**) and RMBMs (**P**), and absolute number of HSCs (**I**) in BM. **C**, **E**, **F** Absolute value of frequencies. Ratio of frequencies of PB B220^+^ cells (**D**), spleen B220^+^ cells (**F**), and BM B220^+^ cells (**H**) in DR mice at each time point to the average frequencies in AL mice. Representative FACS Plots of HSCs (**K**) and CMPs/GMPs/MEPs (**O**). Bone trabecular area in the femoral head, the femoral midshaft and the femoral end. **Q**, **S**, **U** Quantitative analysis of areas and **R**, **T**, **V** representative H&E staining images (scale bar: 100 μm) at indicated time points post-irradiation and no irradiation. **W** and **X** Quantitative analysis of fat droplet area ratio in the femoral end and representative oil red O staining images (scale bar: 100 μm) at indicated time points post-irradiation and no irradiation. Results were displayed as mean ± SD; ns, not significant; *p < 0.05; **p < 0.01; ***p < 0.001; ****p < 0.0001 by Two-way ANOVA test with Tukey’s multiple comparisons test. BM bone marrow, PB peripheral blood, HSC hematopoietic stem cell, CMPs common myeloid progenitors, GMPs granulocyte-macrophage progenitors, MEPs megakaryocyte-erythrocyte progenitors, BMRMs bone marrow resident macrophages. The purple symbols indicate statistical significance between the non-irradiated groups. The green symbols indicate statistical significance between the irradiated groups.

To investigate the effects of post-irradiation DR on hematopoiesis, we used a mouse model where mice were subjected to 4.5 Gy irradiation followed by a DR diet (70% of the intake of age- and sex-matched AL fed mice) (Fig. 1A). We first examined the effects of DR on body weight in mice (Fig.1B). Under both non-irradiation and irradiation conditions, the body weight ratio of mice comparing to the initial timepoint increased in AL group, while it decreased in the DR group over time (Fig. 1B). In the DR group, the median body weight ratio was 0.913 under NIR condition and 0.888 under irradiated condition, indicating a median decrease of 8.7% in the NIR DR mice and 11.2% in IR DR mice. Notably, the maximum weight loss observed was <20% in all the groups, and we observed no significant deterioration in the general health of the mice (Fig. 1B). Hematological parameters were analyzed by PB cell counting and flow cytometric analysis at various time points post-irradiation. Results indicated that, under irradiation conditions, DR mice showed significantly lower counts of total WBCs, neutrophils, and lymphocytes compared to AL mice from two weeks up to two months post-irradiation (Fig. 1C–H). However, there were no significant effects on RBCs and platelet counts (Fig. 1I and J). The spleen and thymus weights were also significantly lower in DR mice compared to AL mice (Fig. 1K–N). We subsequently performed flow cytometric analysis of PB, spleen, and BM cells to determine their frequencies, and conducted Oil Red O and hematoxylin–eosin (H&E) staining on mouse femurs (Fig. 2A), and BM cell counts were significantly reduced (Fig. 2B). Flow cytometric analysis showed that the frequencies of B cells (B220^+^ cells) in peripheral blood, spleen, and BM were consistently lower in DR mice than in AL mice (Fig. 2C–H). Further studies on the frequencies of HSCs (CD150^+^ CD34^−^ c-Kit^+^ Sca-1^+^lineage^−^ cells) and progenitor cells revealed that the frequencies of HSCs and HSC numbers in DR mice were significantly lower than in AL mice from one month up to 10 months post-irradiation (Fig. 2I–K). Similarly, the frequencies of CMPs (CD16/32^−^CD34^+^c-Kit^+^Sca-1^−^lineage^−^ cells) and GMPs (CD16/32^+^CD34^+^c-Kit^+^Sca-1^−^lineage^−^ cells) in the BM were persistently lower in DR mice (Fig. 2L, M, O). We analyzed MEPs (CD16/32^−^CD34^−^c-Kit^+^Sca-1^−^Lineage^−^ cells) and found that under non-irradiation conditions, DR increased MEP frequency compared with AL feeding. However, following IR, MEP frequency did not differ significantly between DR and AL groups (Fig. 2N, O). In our previous studies, we primarily focused on the effects of DR on the hematopoietic system under non-irradiation conditions. In this study, we again used NIR mice as controls and compared the effects of DR on hematopoiesis under irradiation conditions versus non-irradiation conditions. In consistency with our previous findings, DR led to a decrease in peripheral WBCs, neutrophils, and lymphocyte counts under non-irradiation conditions (Fig. 1C–H), and FACS also revealed a reduction in peripheral lymphocyte counts (Fig. 2C, D). Additionally, a decrease in the weight of lymphoid organs, such as the spleen and thymus (Fig. 1K–N), was observed. However, compared to the NIR group, the DR-induced reduction in peripheral WBCs, neutrophils, and lymphocytes was more pronounced in the irradiated group (Fig. 1C–H). Notably, at the two-month post-irradiation time point, the difference in peripheral WBCs between DR and AL groups was no longer significant in the NIR group (Fig. 1C, D). In contrast, in the irradiated group, the DR group still exhibited significantly lower levels of peripheral white blood cells compared to the AL group (Fig. 1C, D). Similarly, nitrification and lymphocyte counts (Fig. 1E–H), as well as the proportion of lymphocytes in the PB, spleen, and BM (Fig. 2C–H), remained significantly lower in the DR group compared to the AL group in the irradiated group at the two-month time point, with a greater magnitude of reduction than that observed in the NIR groups (Figs. 1E–H, 2C–F). These results suggest that DR significantly inhibits hematopoiesis post-irradiation, with this inhibitory effect being sustained over a relatively extended period and affecting various hematopoietic levels from HSCs to downstream peripheral lymphoid organs and PB cell counts.

### Post-irradiation DR disturbs recovery of bone marrow environment

We further investigated the effects of DR on the BM environment. BMRMs which are located within the mesenchymal niche, play a crucial role in retaining HSCs within their niche, maintaining their quiescence, and protecting them from oxidative stress [36]. We examined frequencies of BMRMs by FACS analysis. In NIR mice, there was no significant difference in BMRMs between the AL and DR groups (Fig. 2P). However, at day 14 post-irradiation, a significant reduction in BMRMs was observed in both AL and DR mice (Fig. 2P). During the post-irradiation period, BMRMs in the AL group gradually recovered, returning to pre-irradiation levels by 2 months post-irradiation (Fig. 2P). In contrast, the DR group exhibited a sustained reduction in BMRMs (Fig. 2P). These findings suggest that post-irradiation DR can significantly suppress the recovery of BMRMs.

Previous studies have shown that irradiation leads to significant reductions in bone trabeculae area and increases in fat droplets in BM [37], which are closely related to reduced HSC and hematopoietic cell numbers and function. Osteoblasts in the femur are known to promote hematopoiesis, and one result of osteoblast activity is increased bone trabeculae [17]. To investigate the impact of DR on the BM environment, we performed BM sections of femurs from DR and AL mice under non-irradiation conditions as well as irradiation conditions, and compared bone trabeculae in the femoral head, femoral midshaft, and femoral end (Fig. 2Q–V). DR showed a neutral effect on bone trabeculae and fat droplets in NIR mice (Fig. 2W, X). Notably, we found that bone trabeculae in IR AL mice were relatively continuous and intact, while those in IR DR mice were thinner and discontinuous. Quantitative analysis revealed that the bone trabeculae area in IR DR mice was significantly lower than in IR AL mice at 14 days and one month post-irradiation (Fig. 2Q–V). Oil red O staining further showed that the fat droplet area ratio was significantly higher in IR DR mice at all time points (Fig. 2W, X), suggesting that IR DR exacerbates the reduction of bone trabeculae area and fat droplet accumulation post-irradiation, which may underlie the inhibition of hematopoiesis.

### Post-irradiation DR impairs regeneration capacity of HSCs

**Fig. 3:**
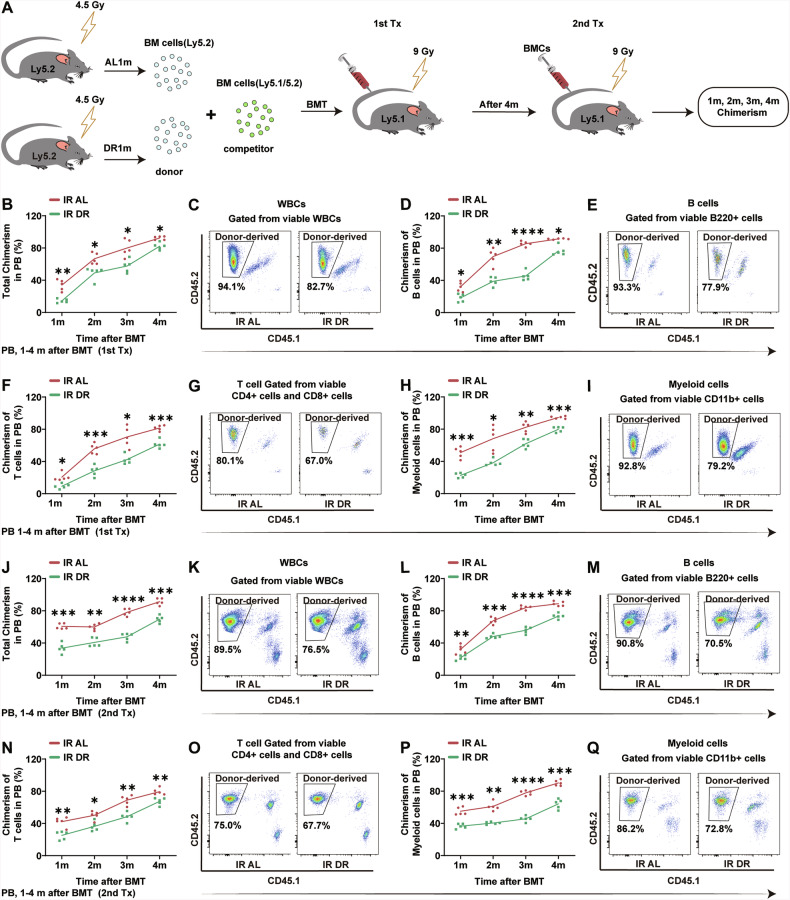
DR post-irradiation suppresses hematopoietic cell function in mice. **A** Experimental scheme. Two-month-old wild-type C57BL/6 mice were subjected to 4.5 Gy X-ray irradiation and subsequently fed with an AL diet or DR diet for one month. Afterwards, 2 × 10⁶ BM cells from irradiated Ly5.2 donor mice were mixed with 1 × 10⁶ BM cells from Ly5.1/5.2 heterozygous competitor mice and primarily transplanted into lethally irradiated (9 Gy) Ly5.1 recipient mice for competitive BM transplantation. Four months after the primary transplantation, 10 × 10^6^ BM cells from the primary recipients were serially transplanted into lethally irradiated (9 Gy) Ly5.1 recipient mice. PB chimerism was analyzed at specified time points post-transplantation via FACS using blood collected from the retro-orbital sinus (*n* = 5 mice per group from 1 experiment representative of 2 independent experiments). **B**–**I** Chimerism of donor-derived cells in the peripheral blood, including WBCs (**B**), B cells (**D**), T cells (**F**), and myeloid cells (**H**), after primary transplantation at the indicated time points. Representative FACS plot of WBCs (**C**), B cells (**E**), T cells (**G**), and myeloid cells (**I**) 4 months after primary transplantation. Chimerism of donor-derived cells in the PB, including WBCs (**J**), B cells (**L**), T cells (**N**), and myeloid cells (**P**), after secondary transplantation at the indicated time points. Representative FACS plot of WBCs (**K**), B cells (**M**), T cells (**O**), and myeloid cells (**Q**) 4 months after secondary transplantation. Results were displayed as mean ± SD;. ns, not significant; *p < 0.05; **p < 0.01; ***p < 0.001; ****p < 0.0001 by Two-way ANOVA test with Tukey’s multiple comparisons test. BMT: bone marrow transplantation.

To determine the effect of DR on hematopoietic regeneration function per se post-irradiation, we performed BM transplantation experiments. BM cells from DR mice at one month post-irradiation were mixed with competitor BM cells at a 2:1 ratio and transplanted into recipient mice. Donor-derived cell chimerism was analyzed at different time points (1 month, 2 months, 3 months and 4 months) post-transplantation (Fig. 3A). We found that donor-derived cell chimerism was significantly lower in DR mice than in AL mice at all time points after primary transplantation (Fig. 3B–I). We further conducted a secondary transplantation experiment, and the results showed that BM from irradiated DR mice exhibited a poorer engraftment rate in the secondary transplantation (Fig. 3J, K). Further analysis revealed that the engraftment rates of PB B cells, T cells, and myeloid cells were also lower in irradiated DR mice compared to the AL group (Fig. 3L–Q), indicating that post-irradiation DR significantly impairs the regeneration capacity of BM hematopoietic cells per se.

### Post-irradiation DR impairs DNA repair and leads to persistent DNA damage response in hematopoietic cells

**Fig. 4:**
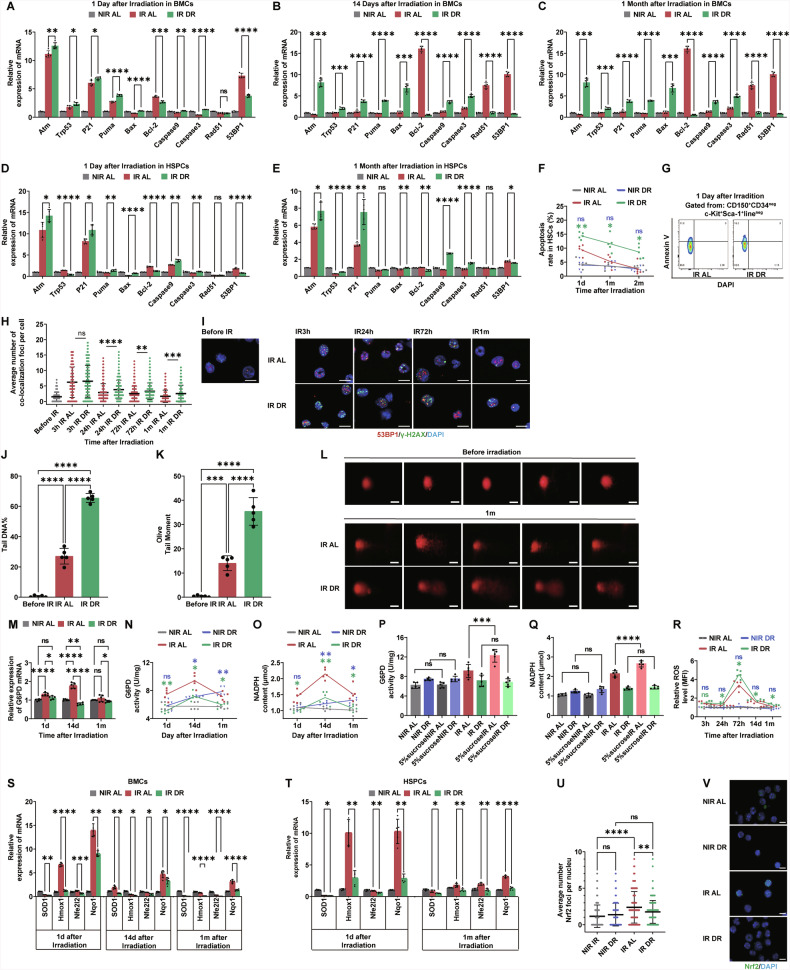
Post-irradiation DR inhibits DNA repair and PPP and leads to persistent DNA damage response in hematopoietic cells. **A**–**E** Mice were subjected to 4.5 Gy X-ray irradiation and subsequently fed with AL diet or DR diet. BM cells or c-Kit^+^ HSPCs were harvested at the indicated time points for q RT-PCR analysis. NIR mice receiving an AL diet were monitored as a control. Relative expression of indicated genes involving DNA damage and repair were analyzed, with β-actin as the internal control (*n* = 5 mice per group from 1 experiment representative of 2 independent experiments). **F**, **G** Apoptosis rates in HSCs at indicated time points post-irradiation and in NIR mice, as determined by FACS (**F**). Representative FACS plots at day 1 post-irradiation (**G**) (*n* = 5 mice per group from 1 experiment, representative of 2 independent experiments). **H**, **I** Quantification and representative images of γH2AX and 53BP1 colocalization in c-Kit^+^ HSPCs at indicated time points post-irradiation and before irradiation, determined by immunofluorescent staining (scale bar: 10 μm) (*n* = 5 mice per group from 1 experiment representative of 2 independent experiments). **J**–**L** Quantification and representative images of comet assay tail DNA in c-Kit^+^ HSPCs at indicated time points post-irradiation and before irradiation (scale bar: 10 μm) (*n* = 5 mice per group from 1 experiment, representative of 2 independent experiments). **M** Relative expression of G6PD mRNA in BM cells of AL and DR mice at the indicated time points post-irradiation and NIR AL mice, analyzed by qRT-PCR with β-actin as the internal control (*n* = 5 mice per group from 1 experiment representative of 2 independent experiments). Dynamic measurements by ELISA of G6PD enzyme activity (**N**) and NADPH (**O**) content in BM cells of AL and DR mice at the indicated time points under IR or no-conditions conditions. **P** G6PD enzyme activity and **Q** NADPH content in BM cells of AL and DR mice given 5% sucrose water or normal drinking water at NIR/IR 14 days (*n* = 5 mice per group from 1 experiment representative of 2 independent experiments). **R** Average fluorescence intensity of ROS in BM cells of AL and DR mice at indicated time points under non-irradiated or irradiated conditions (*n* = 5 mice per group from 1 experiment representative of 2 independent experiments). Relative expression of indicated genes involving ROS in BM cells (**S**) and c-Kit^+^ HSPCs (**T**) of AL and DR mice at indicated time points post-irradiation, analyzed by qRT-PCR with NIR AL mice as a control (*n* = 5 mice per group from 1 experiment representative of 2 independent experiments). **U** and **V** Quantification and representative images of Nrf2 foci in the nucleus of BM cells of AL and DR mice at 5 h post-irradiation and under non-irradiated conditions, determined by immunofluorescent staining (scale bar: 10 μm) (*n* = 5 mice per group from 1 experiment representative of 2 independent experiments). Results were displayed as mean ± SD;. ns, not significant; *p < 0.05; **p < 0.01; ***p < 0.001; ****p < 0.0001 by one-way ANOVA test and Two-way ANOVA test with Tukey’s multiple comparisons test. HSPCs: hematopoietic stem and progenitor cells. The purple symbols indicate statistical significance between the non-irradiated groups. The green symbols indicate statistical significance between the irradiated groups.

Previous studies have shown that irradiation, even at low doses, can significantly activate the DNA damage signaling pathway in HSCs [38, 39], thereby affecting their hematopoietic function. To investigate the mechanism by which DR influences the hematopoietic function of HSCs post-irradiation, we first monitored the DNA damage signaling pathway in hematopoietic cells. Using qRT-PCR, we examined the expression of various genes involved in the DNA damage signaling pathway. We found that DR significantly upregulated the expression of DNA damage-related genes *Atm, Trp53, P21, Puma, Bax, caspase9* and *caspase3* in both total BM cells and HSPCs (Fig. 4A–E). Concurrently, the expression of the anti-apoptotic signal molecule *Bcl-2* was downregulated (Fig. 4A–E). The primary forms of DNA damage induced by ionizing radiation include single-strand breaks (SSBs) and double-strand breaks (DSBs), sugar and base modifications, base oxidation damage, interstrand crosslinks, DNA-protein crosslinks, and locally multiply damaged sites (LMDS) [40]. Among these, DSBs are considered the most lethal form of DNA damage [27], as unrepaired or improperly repaired DSBs can lead to cell death or carcinogenesis, thereby affecting cell fate. DSBs are repaired by NHEJ or HR HSCs predominantly use non-homologous end joining (NHEJ) to repair DSBs caused by ionizing radiation [41, 42]. We further examined the expression of *53BP1* (p53 binding protein 1) and *Rad51* (Rad51 recombinase), molecules related to the DNA damage repair signaling pathway, by qRT-PCR. We found that DR significantly downregulated the expression of *53BP1*, a key molecule in NHEJ, while the expression of *Rad51*, a gene involved in homologous recombination repair, was significantly downregulated in the early stages post-irradiation (Fig. 4A–E). Our experiments showed that DR significantly upregulated the DNA damage signaling pathway in irradiated hematopoietic cells, leading to sustained activation of DDR. The expression of 53BP1, which is involved in DNA damage repair, remained suppressed for extended periods post-irradiation. FACS analysis revealed that apoptosis in HSCs was consistently higher in DR mice compared to AL-fed irradiated mice, while it was similar in DR and AL NIR mice (Fig. 4F, G). Irradiation-induced DSBs trigger phosphorylation of H2AX at serine 139, forming γH2AX foci. In response to IR, 53BP1 becomes hyperphosphorylated and colocalizes with γH2AX, participating in protein recruitment, signal transduction, and repair pathway choice, making simultaneous detection of γH2AX and 53BP1 colocalization an effective approach for analyzing DSB formation and repair [43]. To elucidate the effect of DR on DNA damage and its repair in hematopoietic cells, we sorted c-Kit^+^ hematopoietic cells and performed immunofluorescence staining for γ-H2AX and 53BP1 colocalization and counting at different time points post-irradiation (Fig. 4H, I). γH2AX and 53BP1 colocalization was hardly detectable in NIR mice (Fig. 4H, I). Both the AL and DR groups showed upregulated γH2AX and 53BP1 colocalization at the early time point (3 h) post-irradiation. However, γH2AX and 53BP1 colocalization significantly decreased in AL mice 24 h after irradiation, while they remained elevated in DR mice. At 72 h, γH2AX and 53BP1 colocalization further decreased in AL mice but stayed high in DR mice. Throughout the early time points and up to 72 h, γH2AX and 53BP1 colocalization was consistently higher in DR mice. After one month, γH2AX and 53BP1 colocalization in AL mice had significantly decreased to about one foci per cell, whereas DR mice still exhibited approximately two foci per cell, indicating inhibited DNA damage repair due to DR. This suggests that post-irradiation DR significantly suppresses DNA damage repair. We further assessed DNA damage in hematopoietic cells one month post-irradiation using the Comet assay, which also showed that DR mice exhibited significantly higher levels of DNA damage comparing to AL mice (Fig. 4J–L). In NIR mice, tail DNA and Olive tail moment were at extremely low levels (Fig. 4J–L). These results indicate that DR significantly suppresses DNA damage repair after irradiation in hematopoietic cells.

### Post-irradiation DR significantly inhibits PPP in hematopoietic cells

The above experimental results indicate that post-irradiation DR significantly suppresses DNA damage repair in hematopoietic cells. To further investigate the underlying mechanisms, we examined the PPP. Recent studies have shown that DR under homeostatic conditions can significantly inhibit the glycolytic metabolic pathway in hematopoietic cells [44]. The PPP synthesizes the pentose phosphates required for DNA synthesis during DNA damage repair. The PPP is another major pathway for glucose metabolism. Radiation exposure directly causes DNA damage and also generates a large amount of ROS, which can further lead to DNA damage [45]. NADPH produced by the PPP is crucial for combating ROS and repairing ROS-induced damage [46]. Additionally, the PPP provides the necessary precursor R5P for DNA damage repair [47]. We studied the regulatory effect of post-irradiation DR on the PPP.

Firstly, we performed qRT-PCR to detect the expression of G6PD in BM cells of DR mice at different time points post-irradiation (Fig. 4M). G6PD is the rate-limiting enzyme of the PPP, catalyzing the conversion of glucose-6-phosphate to 6-phosphoglucono-δ-lactone, which is the first and rate-limiting step of the PPP. We found that post-irradiation, the expression of G6PD was significantly upregulated in the BM cells of AL mice, whereas it was not upregulated and was significantly lower in DR mice compared to AL mice (Fig. 4M). This strong inhibitory effect on G6PD expression by DR was observed at 1 day, 14 days, and one month post-irradiation, with the most pronounced effect at 14 days (Fig. 4M). We further measured G6PD enzyme activity using an ELISA method (Fig. 4N). Similarly, at 1 day, 14 days, and one month post-irradiation, G6PD activity in DR mice was significantly lower than in AL mice. NADPH, a major product of the PPP, primarily originates from the PPP and reflects its activity. Therefore, we further assessed NADPH levels to validate the expression regulation of the PPP in whole BM cells post-irradiation and the effect of DR (Fig. 4O). We found that NADPH levels were significantly lower in DR mice compared to AL mice at 1 day, 14 days, and one month post-irradiation (Fig. 4O). These findings indicate that on 14 days post-irradiation, DR significantly inhibits the PPP in BM hematopoietic cells. Interestingly, in the IR group of mice, we observed that although there was no significant difference in G6PD enzyme activity and NADPH levels between the AL and DR groups at day 3, at days 14 and 30, the NIR DR mice exhibited higher G6PD enzyme activity and NADPH levels compared to the AL group (Fig. 4N, O).

To further investigate the effect on the PPP by DR, we provided mice with high-glucose drinking water and measured G6PD and NADPH levels after 14 days (Fig. 4P, Q). In irradiated AL mice, high glucose further increased G6PD enzyme activity and NADPH levels. However, in irradiated DR mice, there was no significant upregulation of G6PD activity or NADPH levels even after high-glucose supplementation, indicating that DR significantly inhibits PPP activation post-irradiation. However, in the NIR mice, a high-sugar diet did not significantly increase G6PD enzyme activity or NADPH levels in either the AL or DR groups (Fig. 4P, Q).

We also measured the relative fluorescence intensity of ROS using FACS, finding that DR mice had consistently higher ROS levels compared to AL mice at the one-month time point (Fig. 4R). Additionally, qRT-PCR analysis of antioxidant gene expression in BM cells and c-Kit^+^ cells revealed that *SOD1, Hmox1, Nfe2l2* and *Nqo1* gene expression levels were consistently lower in DR mice compared to AL mice at the one-month time point (Fig. 4S, T). This indicates that DR significantly increases ROS levels and reduces ROS scavenging. When intracellular ROS levels increase (e.g., during oxidative stress), the critical cysteine residues in Keap1 are oxidized or modified, leading to the release of Nrf2 from the Keap1 complex, thereby escaping degradation and translocating into the nucleus [48, 49]. Nrf2 plays a pivotal role in responding to oxidative stress (elevated ROS), inflammation, toxins, and radiation [50]. We assessed Nrf2 expression using immunofluorescence staining. In the NIR groups, both AL and DR mice exhibited extremely low levels of Nrf2 expression, with no significant difference between the two groups (Fig. 4U, V). However, at 5 hours post-irradiation, a marked increase in Nrf2 nuclear translocation and expression was observed in AL mice (Fig. 4U, V), whereas DR mice showed no significant upregulation of Nrf2 or nuclear translocation (Fig. 4U, V). A comparison between the irradiated AL and DR groups revealed that Nrf2 expression levels in DR mice were significantly lower than those in AL mice (Fig. 4U, V). These findings suggest that DR treatment significantly suppresses Nrf2 upregulation and nuclear translocation following irradiation, thereby impairing the activation of the antioxidant stress pathway and preventing effective ROS clearance.

### Down-regulation of the PPP impairs hematopoiesis in AL mice post-irradiation

**Fig. 5:**
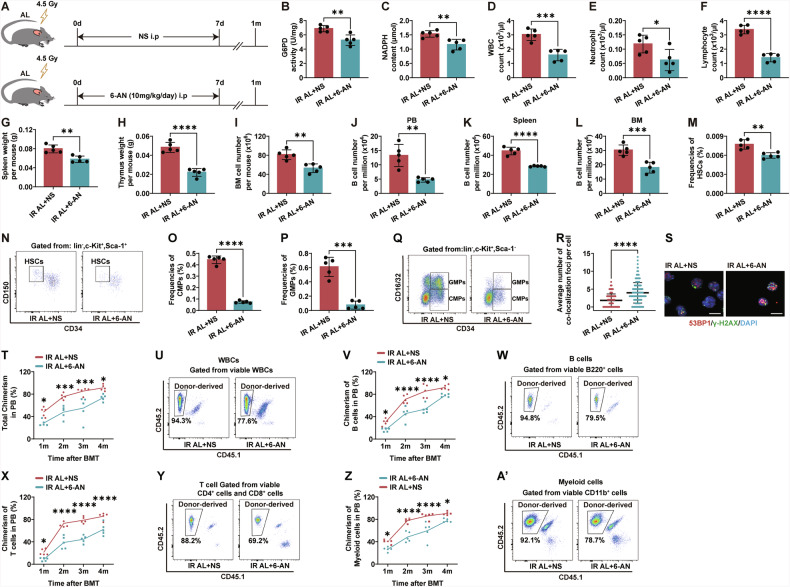
Inhibition of the PPP suppresses the hematopoietic system in irradiated mice. **A** Experimental scheme. Wild-type C57BL/6 mice (2 months old) were irradiated with 4.5 Gy X-rays and fed with an AL diet. Mice were then injected intraperitoneally with 10 mg/kg of 6-aminonicotinamide (6-AN) or saline daily for 7 days post-irradiation, and were continued on an AL diet until 1 month post-irradiation (*n* = 5 mice per group from 1 experiment representative of 2 independent experiments). G6PD enzyme activity (**B**) and NADPH content (**C**) in BM cells of AL mice 1 month post-irradiation, analyzed using ELISA. WBC (**D**), Neutrophil (**E**), and lymphocyte (**F**) counts in PB of AL mice 1 month post-irradiation. **G**, **H** Spleen and thymus weights of AL mice 1 month post-irradiation. **I** Total BM cell counts of AL mice 1 month post-irradiation. FACS analysis of frequencies of B220^+^ lymphocytes in PB (**J**), spleen (**K**), BM (**L**); and frequencies of HSCs (**M**), CMPs (**O**), and GMPs (**P**), and representative FACS Plots of HSCs (**N**) and CMPs/GMPs/MEPs (**Q**) of AL mice 1 month post-irradiation. **R** and **S** Quantification and representative images of γH2AX and 53BP1 colocalization in BM cells of AL mice 1 month post-irradiation, determined by immunofluorescent staining (scale bar: 10 μm). **T–A’** Chimerism of donor-derived cells in the PB, including WBCs (**T**), B cells (**V**), T cells (**X**), and myeloid cells (**Z**) chimerism after transplantation at indicated time points and representative FACS plot of WBCs (**U**), B cells (**W**), T cells (**Y**), myeloid cells (**A’**) at 4 months post-transplantation. Results were displayed as mean ± SD;. ns, not significant; *p < 0.05; **p < 0.01; ***p < 0.001; ****p < 0.0001 by unpaired two-tailed Student’s *t*-test and Two-way ANOVA test with Tukey’s multiple comparisons test. IR AL + 6-AN: irradiated ad libitum group injected with 6-AN; IR AL + NS: irradiated ad libitum group injected with saline.

To further validate the impact of the PPP on DNA repair and hematopoietic function in irradiated hematopoietic cells, we administered 6-AN injections to AL mice post-irradiation (Fig. 5A). 6-AN is a PPP inhibitor that, once captured in mammalian cells, is converted by NAD(P) glycohydrolase to the non-reducible forms 6-ANAD and 6-ANADP, with 6-ANADP being a strong competitive inhibitor of G6PD [51, 52]. We administered 6-AN immediately after irradiation, while control mice received saline injections. The injections were continued for seven consecutive days. One month later, we harvested the total BM cells to measure G6PD enzyme activity and NADPH levels. The 6-AN injections significantly reduced G6PD activity and NADPH levels in the BM cells of AL mice (Fig. 5B, C). Interestingly, we observed that post-irradiation 6-AN administration significantly decreased PB WBCs, neutrophils, and lymphocytes (Fig. 5D–F). Additionally, the weights of the spleen and thymus were significantly reduced in the 6-AN group (Fig. 5G, H). The total number of BM cells was markedly lower in the 6-AN injection group compared to the control group (Fig. 5I). Further FACS analysis showed a significant decrease in the frequencies of B cells in the PB, spleen and BM of the 6-AN group (Fig. 5J–L). The frequencies of HSCs and progenitor cells, including CMPs and GMPs, were also significantly reduced in the 6-AN group (Fig. 5M–Q). We further assessed the effect of PPP inhibition on DNA damage repair using immunofluorescence staining for γ-H2AX and 53BP1 colocalization. We found that the number of γ-H2AX and 53BP1 colocalization in the BM of the 6-AN group was significantly higher than in the saline-injected control group, indicating that 6-AN inhibits DNA damage repair (Fig. 5R, S). To explore the effect of PPP inhibition and DNA damage repair suppression on hematopoietic function, we harvested whole BM cells from the mice at the one-month time point and performed hematopoietic cell competitive transplantation. We assessed donor-derived chimerism at different time points post-transplantation. The chimerism of donor-derived cells, including total chimerism, B cells, T cells, and myeloid cells, was significantly lower in the 6-AN group compared to the saline-injected control group (Fig. 5T–A’). These results further demonstrate that inhibiting the PPP with 6-AN impairs the hematopoietic cell reconstitution capacity in irradiated AL mice.

### UP-regulation of the PPP rescues hematopoiesis in post-irradiation DR mice

**Fig. 6:**
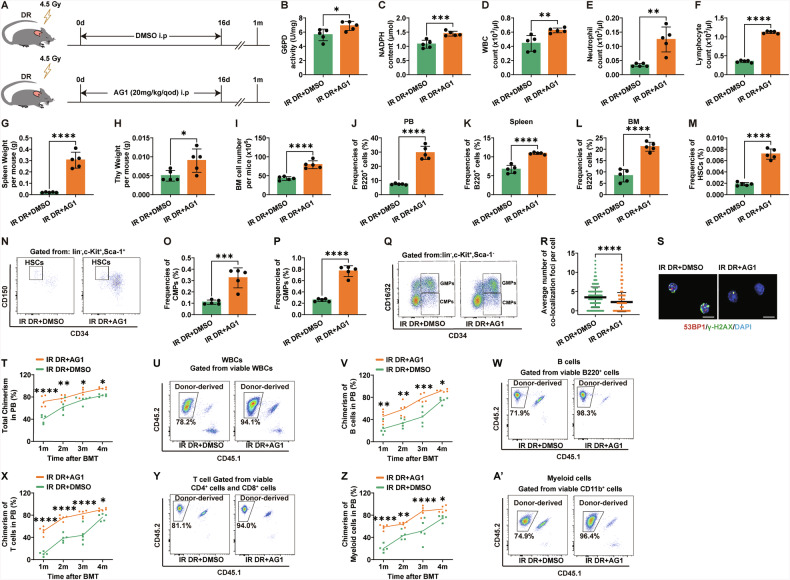
Activation of the PPP improves hematopoietic function in dietary-restricted (DR) mice post-irradiation. **A** Experimental scheme. Wild-type C57BL/6 mice (2 months old) were irradiated with 4.5 Gy X-rays, fed with a DR diet. Mice were then injected intraperitoneally with 20 mg/kg of AG1 or DMSO every other day for 9 times post-irradiation, and were continued on DR until 1 month post-irradiation (*n* = 5 mice per group from 1 experiment representative of 2 independent experiments). G6PD enzyme activity (**B**) and NADPH content (**C**) in BM cells of DR mice 1 month post-irradiation, analyzed using ELISA. WBC (**D**), Neutrophil (**E**), and lymphocyte (**F**) counts in PB of DR mice 1 month post-irradiation. **G**, **H** Spleen and thymus weights of DR mice 1 month post-irradiation. **I** Total BM cell counts of DR mice 1 month post-irradiation. FACS analysis of frequencies of B220^+^ lymphocytes in PB (**J**), spleen (**K**), BM (**L**); and frequencies of HSCs (**M**), CMPs (**O**) and GMPs (**P**), and representative FACS Plots of HSCs (**N**) and CMPs/GMPs/MEPs (**Q**) of DR mice 1 month post-irradiation. **R** and **S** Quantification and representative images of γH2AX and 53BP1 colocalization in BM cells of DR mice 1 month post-irradiation, determined by immunofluorescent staining (scale bar: 10 μm). Chimerism of donor-derived cells in the PB, including WBCs (**T**), B cells (**V**), T cells (**X**), and myeloid cells (**Z**) chimerism after transplantation at indicated time points and representative FACS plot of WBCs (**U**), B cells (**W**), T cells (**Y**), myeloid cells (**A’**) at 4 month post-transplantation. Results were displayed as mean ± SD;. ns, not significant; *p < 0.05; **p < 0.01; ***p < 0.001; ****p < 0.0001 by unpaired two-tailed Student’s *t* test, and Two-way ANOVA test with Tukey’s multiple comparisons test. IR DR + AG1: irradiated dietary restriction group injected with AG1; IR DR + DMSO: irradiated dietary restriction group injected with DMSO.

To further elucidate the regulatory role of PPP in DR mice post-irradiation, we activated PPP using the activator AG1 in DR mice post-irradiation (Fig. 6A). G6PD is functionally active in dimeric or tetrameric forms. AG1 promotes the formation and stabilization of G6PD dimers, thereby enhancing G6PD activation [53, 54]. In vivo experiments in mice have confirmed the stimulatory effect of AG1 on PPP [53, 54]. We first confirmed successful activation of PPP by detecting increased G6PD enzyme activity and NADPH levels in BM cells of DR mice by AG1 injection (Fig. 6B, C). AG1 administration also significantly improved hematopoietic parameters, including WBC, neutrophil, and lymphocyte counts in PB (Fig. 6D–F). Additionally, spleen and thymus weights were significantly higher in AG1-treated mice compared to the control group (Fig. 6G, H). The total BM cell count was increased in the AG1 injection group (Fig. 6I). FACS analysis revealed that the frequencies of B cells in the PB, spleen and BM were significantly higher in the AG1 group compared to the DMSO group (Fig. 6J–L). Additionally,. The frequencies of HSCs, CMPs, and GMPs were also significantly higher in the AG1 group compared to the DMSO control group (Fig. 6M–Q). These experiments indicate that activating the PPP with AG1 in post-irradiation DR mice can promote hematopoietic regeneration.

**Fig. 7:**
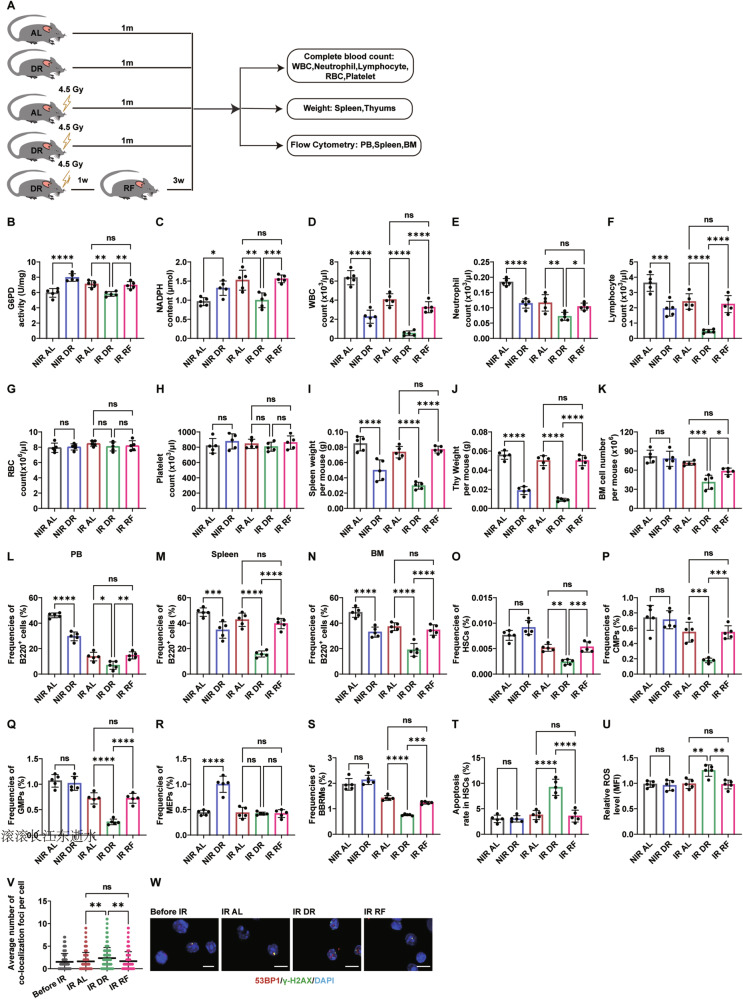
Suppression of hematopoiesis in mice by RF post-irradiation. **A** Experimental scheme. Wild-type C57BL/6 mice (2 months old) were irradiated with 4.5 Gy X-rays, fed with either AL diet or DR diet or DR diet 1 week, then AL feeding 3 weeks (daily food intake restricted to 70% of the intake of age- and sex-matched AL mice). NIR mice receiving an AL or DR diet were also monitored as a control (*n* = 5 mice per group from 1 experiment representative of 2 independent experiments. **B** and **C** G6PD enzyme activity (**B**) and NADPH content (**C**) in BM cells of RF mice 1 month post-irradiation, analyzed using ELISA. WBC (**D**), Neutrophil (**E**), and lymphocyte (**F**), RBC (**G**), platelet (**H**) counts in PB of RF mice 1 month post-irradiation. **I**, **J** Spleen and thymus weights of RF mice 1 month post-irradiation. **K** Total BM cell counts of RF mice 1 month post-irradiation. FACS analysis of frequencies of B220^+^ lymphocytes in PB (**L**), spleen (**M**), BM (**N**); and frequencies of HSCs (**O**), CMPs (**P**) and GMPs (**Q**), MEPs (**R**), and RMBMs (**S**) of RF mice 1 month post-irradiation. **T** Apoptosis rates in HSCs of RF mice 1 month post-irradiation. **U** Average fluorescence intensity of ROS in BM cells of RF mice 1 month post-irradiation. **V** and **W** Quantification and representative images of γ-H2AX and 53BP1 colocalization in BM cells of RF mice 1 month post-irradiation, determined by immunofluorescent staining (scale bar: 10 μm). Results were displayed as mean ± SD; ns, not significant; *p < 0.05; **p < 0.01; ***p < 0.001; ****p < 0.0001 by one-way ANOVA test with Tukey’s multiple comparisons test. IR RF: irradiated refeeding group.

We further investigated the effect of AG1 on DNA damage repair in hematopoietic cells. Immunofluorescence staining for γ-H2AX and 53BP1 colocalization showed that the number of γ-H2AX and 53BP1 colocalized foci in BM cells was significantly reduced in the AG1 injection group (Fig. 6R, S), suggesting that AG1 activation of the PPP can significantly improve DNA damage repair in hematopoietic cells of post-irradiation dietary-restricted mice. To further clarify its impact on hematopoietic reconstitution, we transplanted BM cells from one-month-old mice with competitor cells into recipient mice. We found that the hematopoietic reconstitution capacity of the AG1 activation group was significantly higher than that of the DMSO control group (Fig. 6T–A’). The total chimerism, B cell chimerism, T cell chimerism, and myeloid cell chimerism in the PB of the AG1 group were significantly higher than those in the DMSO group (Fig. 6T–A’). These experiments indicate that upregulating the PPP with AG1 significantly improves DNA damage repair and promotes hematopoietic reconstitution in hematopoietic cells of post-irradiation DR mice.

### Post-irradiation DR followed by RF significantly promotes hematopoiesis

To further examine the effects of refeeding (RF) on DNA repair and hematopoietic function after irradiation, mice were subjected to DR for one week, followed by three weeks of AL feeding (Fig. 7A). RF significantly increased G6PD activity and NADPH levels in BM cells (Fig. 7B and C). At one month post-irradiation, RF mice exhibited higher WBC, neutrophil, and lymphocyte counts than DR mice, comparable to AL controls, while RBC and platelet counts were unaffected (Fig. 7D–H). Spleen and thymus weights and total BM cell count were significantly elevated in RF mice (Fig. 7I–K). Flow cytometric analysis revealed increased frequencies of B cells, HSCs, CMPs, GMPs, and BMRMs, but not MEPs, in RF mice compared with DR mice (Fig. 7L–S). HSC apoptosis was reduced in RF mice to levels similar to AL controls (Fig. 7T), and ROS levels were lower than in DR mice (Fig. 7U). Immunofluorescence staining for γ-H2AX and 53BP1 colocalization showed fewer colocalized foci in RF BM cells than in DR mice, indicating enhanced DNA repair (Fig. 7V and W). These results indicate that short-term DR following radiation exposure can induce hematopoietic suppression and an increase in DNA damage, but these phenotypes can be reversed upon resumption of ad libitum feeding.

### Severe neutropenia negatively relates to BMI in patients receiving pelvic radiotherapy

**Fig. 8:**
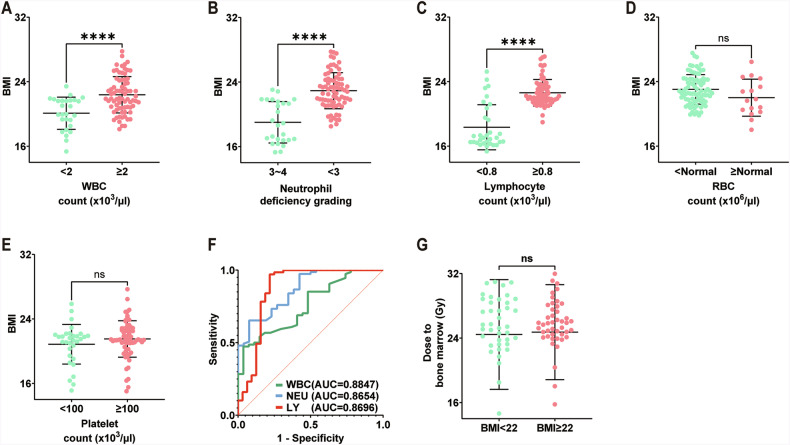
Lower-weight individuals are more likely to have haematopoietic damage after pelvic radiotherapy. Height and weight were measured weekly in 101 patients undergoing pelvic radiotherapy, and BMI was calculated by taking the mean of the BMI values of the same patient. The values of PB neutrophil, WBC, lymphocyte, RBC, and platelet counts of the patients were recorded weekly for one month after radiotherapy. **A** Those with a WBC count ≤2 × 10^3^/μL had a smaller BMI than those with a WBC count >2 × 10^3^/μL. **B** Those with neutrophil deficiencies of grade 3–4 have a smaller BMI than those with neutrophil deficiencies less than grade 3. **C** Those with a lymphocyte count ≤0.8 × 10^3^/μL had a smaller BMI than those with a lymphocyte count >0.8 × 10^3^/μL. **D** There was no difference in BMI between those with RBC counts less than or equal to normal and those with RBC counts greater than normal. **E** There was no difference in BMI between those with platelet counts ≤100 × 10^3^/μL and those with platelet counts >100 × 10^3^/μL. **F** ROC curves of PB WBCs, neutrophils, and lymphocytes according to the BMI values corresponding to the above groupings. **G** There is no difference in dose to the BM between patients with high and low BMI. Results were displayed as mean ± SD. ns, not significant; *p < 0.05; **p < 0.01, ***p < 0.001, ****p < 0.0001 by unpaired two-tailed Student’s *t* test. BMI: body mass index.

In order to further study the role of post-irradiation nutritional status on hematopoietic cell counts in patients, we retrospectively collected routine blood counts from 101 patients who received radiotherapy for pelvic cancer at our centre from 2021/10/18 to 2023/6/30. These patients underwent weekly hematological monitoring during radiotherapy, and we recorded WBC, neutrophil, lymphocyte, RBC, and platelet counts, as well as the patients’ BMI (Fig. 8A–E). We found that patients with WBC counts ≤2 × 10^3^/µL the BMI values were significantly lower than those with WBC counts never <2 × 10^3^/µL (Fig. 8A). We compared the data of patients who experienced the most severe neutropenia (grade 3–4) with those who either never experienced neutropenia or only experienced grade <3 toxicity. Similarly for patients with grade 3–4 neutropenia had significantly lower BMI values compared to those who never experienced grade 3 or higher neutropenia (Fig. 8B). In lymphocyte counts it was also found that patients presenting with lymphocytes ≤0.8 × 10^3^/µL had significantly lower BMI than patients with lymphocyte counts never <0.8 × 10^3^/µL (Fig. 8C). As for platelet as well as RBC counts, there was no significant difference in BMI values between patients with platelets ≤100 × 10^3^/µL or >100 × 10^3^/µL, or between patients with and without RBC reduction (Fig. 8D, E). Next, we performed the ROC test on the BMI after grouping the WBC, neutrophil and lymphocyte counts, which differed in the random test, and plotted the ROC curve, and calculated the Yoden index to be 21.59 for WBCs, 21.06 for neutrophil, and 20.73 for lymphocytes (Fig. 8F). The results indicated that the degree of myelosuppression after irradiation and the BMI were related. Based on the Youden index calculated from the ROC curve, patients were divided into two groups: BMI ≥ 22 (high BMI group) and BMI < 22 (low BMI group). The basic characteristics of the patients were presented in Supplementary Table 3. A comparison of the BW dose between the two groups revealed no statistically significant difference (Fig. 8G).

## Discussion

Radiation therapy remains one of the main treatments for tumors. However, radiotherapy often involves the pelvis, flat bones, and long bones [55], which are hematopoietic tissues. Even low doses of radiation can cause significant damage to these tissues and reduce blood cell counts [27]. Therefore, hematological toxicity is one of the most common complications of radiotherapy. Severe reductions in WBCs [4] or platelets [56] due to radiotherapy can even lead to treatment interruptions, affecting the overall efficacy and prognosis of anti-tumor treatment. Although numerous studies have demonstrated the negative impact of radiation on hematopoiesis [56, 57], the factors causing severe hematopoietic dysfunction in some patients remain unclear. Our study is the first to propose that food reduction post-irradiation can significantly inhibit hematopoiesis.

We are the first to propose that DR post-irradiation can cause hematopoietic cells to remain at persistently low levels with significantly delayed recovery. BM transplantation experiments further confirmed significant impairment of HSC function. We provide the first experimental evidence that DR post-irradiation can significantly inhibit the activation of PPP in hematopoietic cells, reduce NADPH, thereby inhibiting DNA damage repair and reducing ROS antioxidation. Activating PPP in DR mice can significantly improve HSC function, allowing hematopoiesis to recover, while inhibiting PPP in AL mice, the effects of DR impairing hematopoiesis. Therefore, we mechanistically show that DR post-irradiation impairs DNA damage repair and regeneration capacity of HSCs through downregulation of the PPP, which produces NADPH that plays a crucial role in mitigating ROS [45] and provides necessary 5-phosphoribose for DNA damage repair [58].

Clinically, we found that patients with lower BMI and poorer nutritional status are more likely to experience severe reductions in WBCs, neutrophils, and lymphocytes during pelvic radiotherapy. This is the first clinical proof of a clear correlation between nutritional status and hematological toxicity of radiotherapy. Our study identifies a new mechanism for radiotherapy-induced hematological toxicity and confirms the crucial role of nutritional intake in post-radiation hematopoiesis. Notably, in the clinical setting, patients receiving radiotherapy often undergo concurrent chemotherapy, and radiotherapy itself is usually delivered in fractionated doses over an extended course (for example, standard long-course radiotherapy for rectal cancer lasts approximately five weeks). Moreover, given that patients typically undergo continuous anti-tumor treatment, reductions in dietary intake often persist for several weeks to months, or even longer. In the current study, we investigated the effects of DR lasting up to 2 months on hematopoiesis, which can well reflect the actual clinical situation, and thus may have important clinical implications. The current study indicates a potential role of nutritional status on DNA damage repair and ROS levels through regulation of the PPP, and consequently affecting HSCs and hematopoiesis. The study draws attention to maintaining good nutritional status and dietary intake for patients undergoing radiotherapy to reduce hematopoietic toxicities, so as to ensure continuation of radiotherapy and subsequent treatments, thereby improving the prognosis.

Although the clinical observation of hematologic toxicity related to radiotherapy has been recognized for a long time [1, 2], research into its mechanisms has so far primarily focused on the direct damage to hematopoietic cells caused by radiation, as well as technical modifications in radiotherapy, such as Stereotactic Body Radiotherapy (SBRT) [59] or pelvic (active) BM sparing radiotherapy [60], to minimize the radiation dose to hematopoietic tissues. However, there has been a lack of research on other factors beyond radiation exposure, such as patient-specific factors that might influence hematopoiesis. Our study is the first to identify a clear correlation between a patient’s dietary and nutritional status and radiotherapy-related hematologic toxicity. In this study, we observed that DR following irradiation reduced PB WBCs, neutrophils, and lymphocytes, whereas RBC and platelet counts appeared largely unaffected. Consistent with our previous findings that DR enhanced HSC differentiation toward the megakaryocyte-erythroid lineage under steady-state conditions, these results suggested that while irradiation exerted suppressive effects on erythropoiesis, the intrinsic stimulatory influence of DR on HSC lineage commitment may have partially compensated for radiation-induced inhibition. This interplay could explain why erythropoiesis remained largely comparable between DR and AL groups despite the stress of irradiation. The current study found that DR following radiation exposure can regulate both HSPCs and the BM environment, thereby inhibiting hematopoiesis after radiation exposure.

Radiation exposure leads to significant DNA damage in hematopoietic cells [2, 57], activating the DDR signaling pathway [39], which is one of the primary reasons for radiation-induced impairment of HSC function. This study found that compared to AL, DR can further upregulate genes related to the DDR signaling pathway, including *Atm, P53, P21, Puma, Bax, Caspase9*, and *Caspase3*, while downregulating the expression of the anti-apoptotic signal molecule *Bcl-2*. This regulation of gene expression was observed in both total BM cells and HSPCs. Activation of the DDR signaling pathway simultaneously initiates the DNA damage repair system [38], where the repair of DNA damage in hematopoietic cells can occur through both HR and NHEJ [27]. Among these, NHEJ is the more commonly used repair mechanism in HSPCs [41, 42]. *Rad51* and *53BP1* are key molecules in the HR and NHEJ pathways [41, 61], respectively. Our study found that in irradiated HSPCs of DR mice, the upregulation levels of *Rad51* and *53BP1* were significantly lower than those in AL mice. Using γ-H2AX and 53BP1 colocalized staining and comet assays, we observed a significant delay in DNA damage repair in the HSPCs of DR mice after IR. These findings suggest that DR after radiation exposure inhibits DNA damage repair in the HSPCs of mice, leading to sustained activation of the DDR system and thereby impairing their hematopoietic function. γ-H2AX and 53BP1 colocalized staining and comet assays were performed on c-Kit+ cells, which include proliferative cells (more differentiated progenitors) and quiescent cells (HSCs and less differentiated progenitors). The proportion of HSCs is very low compared to progenitors, so the damaged cells are likely to be almost exclusively progenitors. In BM transplantation experiments, when we transplanted hematopoietic cells from irradiated, DR mice into recipients, the HSCs from the irradiated DR donors showed significantly reduced hematopoietic reconstitution capacity, even though the recipients were fed AL. This further confirms that DR post-irradiation can intrinsically impair the regeneration function of HSCs.

We further explored the mechanism by which DR post-irradiation significantly inhibits DNA damage repair in hematopoietic cells. Previous studies have shown that IR directly causes DNA damage and generates a significant amount of ROS, which can further exacerbate DNA damage [62]. The NADPH produced by PPP is crucial for combating ROS and repairing ROS-induced damage [45]. Additionally, the PPP provides the necessary precursor, R5P, for DNA damage repair [46, 47, 63]. Transgenic mice overexpressing G6PD have been shown to reduce ROS accumulation in multiple systems during aging, thereby extending their lifespan [64]. The current study is the first to discover that irradiation significantly upregulates G6PD and NADPH levels in BM cells and HSPCs of AL mice, indicating activation of PPP. However, DR significantly inhibits the upregulation of G6PD and NADPH. Even high glucose supplementation cannot activate the PPP in HSPCs of DR mice, whereas it further activates the PPP in HSPCs of AL mice. This suggests that DR significantly inhibits the PPP in HSPCs post-irradiation. We also observed that ROS levels in HSPCs of DR mice were significantly higher than those in AL mice post-irradiation. By regulating the PPP, we further revealed its critical role in DNA repair post-irradiation. Based on previous studies, we hypothesize that DR post-irradiation inhibits the activation of PPP, reduces NADPH production, and limits the supply of DNA damage repair substrates. This leads to increased ROS in HSPCs, delayed DNA damage repair, sustained DDR activation, and impaired HSPC function.

Our study also found that DR post-irradiation not only damages hematopoietic cells themselves but also significantly affects the hematopoietic microenvironment. Our research demonstrated that DR post-irradiation can significantly and persistently disrupt the BM microenvironment. DR, administered for both short-term and long-term following irradiation, leads to a significant increase in lipid droplets and a decrease in bone trabeculae in the BM. The increase in lipid droplets and the decrease in bone trabeculae significantly weaken the support for the hematopoietic system [18, 37], thereby exerting a negative regulatory effect on hematopoiesis. This finding is the first to clearly identify the negative regulatory effect of DR on the bone trabeculae and lipid droplets post-irradiation.

Recent studies showed that short-term DR before stress induction, such as chemotherapy and UV radiation, or long-term DR under steady-state conditions, can significantly protect intestinal stem cells, skin, and delay HSC aging [7, 65–67]. The protective effect of pre-stress DR observed in previous research does not contradict the detrimental effect of post-irradiation DR found in the current study. Recent studies show that DR was implemented before the occurrence of stress damage, such as prior to UV irradiation or chemotherapy [65–67]. DR under steady-state conditions (i.e., before the occurrence of damage) can regulate the gut microbiota and initiate a series of protective mechanisms, thereby enhancing resilience against stress when it occurs. Unlike the previous studies, the current study commenced DR only after radiation exposure, without preconditioning the body through DR to establish systemic regulation and protection. Consequently, DR did not exhibit a protective effect on HSCs in this context. A recent publication showed that post-irradiation DR significantly prolongs the quiescent state of hair follicle stem cells and results in higher γH2AX expression in hair follicle cells compared to AL mice [68]. This aligns with our current study’s finding that post-irradiation DR significantly inhibits and delays DNA damage repair in hematopoietic cells. This indicates that the effects of pre-stress DR (i.e., DR under steady-state conditions) and post-stress DR are fundamentally different. Pre-stress (steady-state) DR exhibits protective effects through the regulation of the gut microbiome and other potential mechanisms. In contrast, starting DR after irradiation does not allow sufficient time for the gut microbiome and other possible mechanisms to be regulated before the damage occurs, thus highlighting the inhibitory effect of DR on damage repair. This study complements the previous research by revealing another aspect of DR, showcasing its dual-edged sword effect [17, 69]. We acknowledge that, similar to low BMI, a high BMI may also negatively impact recovery and survival after total body irradiation (TBI), as obese individuals may exhibit altered immune responses and increased systemic inflammation [69]. Investigating these effects in an obese mouse model post-TBI is indeed considered a valuable research direction. In future studies, it will be interesting to use appropriate animal models to explore how both ends of the BMI spectrum—low BMI and high BMI—affect hematopoietic recovery and overall survival after TBI.

The limitations of this study include the fact that, in a clinical setting, radiation therapy for cancer patients aims to minimize damage to normal tissues, including the BM, and patients typically receive myelosuppressive chemotherapy, which was not modeled in our mouse study. The data presented in this study were generated using female C57BL/6J mice. In our mouse model, we used a TBI dose of 4.5 Gy. We confess that TBI is much less often used than targeted region irradiation, which is complicated and difficult in mice in our experimental settings. We also acknowledge that the correlation between doses in animal models and human exposure requires further investigation; thus, caution should be applied when generalizing beyond this scope. The use of mice of the same age and gender in the current study was primarily to reduce variability, as physiological, metabolic, immune, and hematopoietic functions differed significantly between age and gender groups. However, in the real world, patients differ in their age and gender. Therefore, the use of uniform age and gender, as radiation-induced myelosuppression may be influenced by factors such as housing conditions, sex, and genetic background. For example, previous studies indicated that estrogen could promote HSC proliferation [70, 71]. Additionally, anatomical differences and variations in hematopoietic sites between mice and humans, such as the presence of BM in the long bones of mice [72, 73], may affect the translation of these findings to humans.

Furthermore, c-Kit^+^ cells represent a mixture of HSPCs at various stages of differentiation and proliferation. Each cell type may respond differently to radiation. Thus, studying the broad category of c-Kit+ cells as a whole may introduce certain inaccuracies, which we acknowledge as a limitation of our study.

In summary, the current study is the first to discover that DR post-irradiation significantly impairs the function of HSPCs and delays the recovery of BMRMs, osteoblasts and adipocytes in the BM environment. The molecular mechanism was revealed to be the inhibition of DNA damage repair in c-Kit^+^ cells through the suppression of PPP activation. This research suggests that the dietary and nutritional status of radiotherapy patients is closely related to radiotherapy-associated hematologic toxicity, providing new insights into addressing radiotherapy-induced BM suppression. It offers potential solutions, particularly for improving refractory hematopoietic disorders associated with radiotherapy. For patients with low BMI, we recommend consultation with a nutritionist to explore methods for enhancing nutritional management and increasing body weight. Potential interventions may include introducing easily digestible and absorbable nutritional supplements and foods, utilizing intravenous nutritional support, and incorporating probiotics to promote digestion and absorption. These approaches could potentially ameliorate hematopoietic toxicity of low BMI patients induced by radiotherapy of low BMI patients to radiotherapy.

## Supplementary information

Supplementary Table titles

Supplementary Table 1. Detailed nutritional analysis values of mouse diet (per kilogram content)

Supplementary Table 2. Primer sequences

Supplementary Table 3. Baseline demographics, disease characteristics, and irradiation doses to pelvic bone marrow of patients (*n* = 101)

## Data Availability

All data needed to evaluate the conclusions in the paper are present in the paper and/or the Supplementary Materials.
