# Supplementary figures and images for: Post-irradiation dietary restriction impairs hematopoiesis via inhibition of the pentose phosphate pathway in hematopoietic stem and progenitor cells
Source: Cell Death Dis. 2026 Jan 7;17(1):8. doi: 10.1038/s41419-025-08249-w (PMC12779998; doi:10.1038/s41419-025-08249-w)

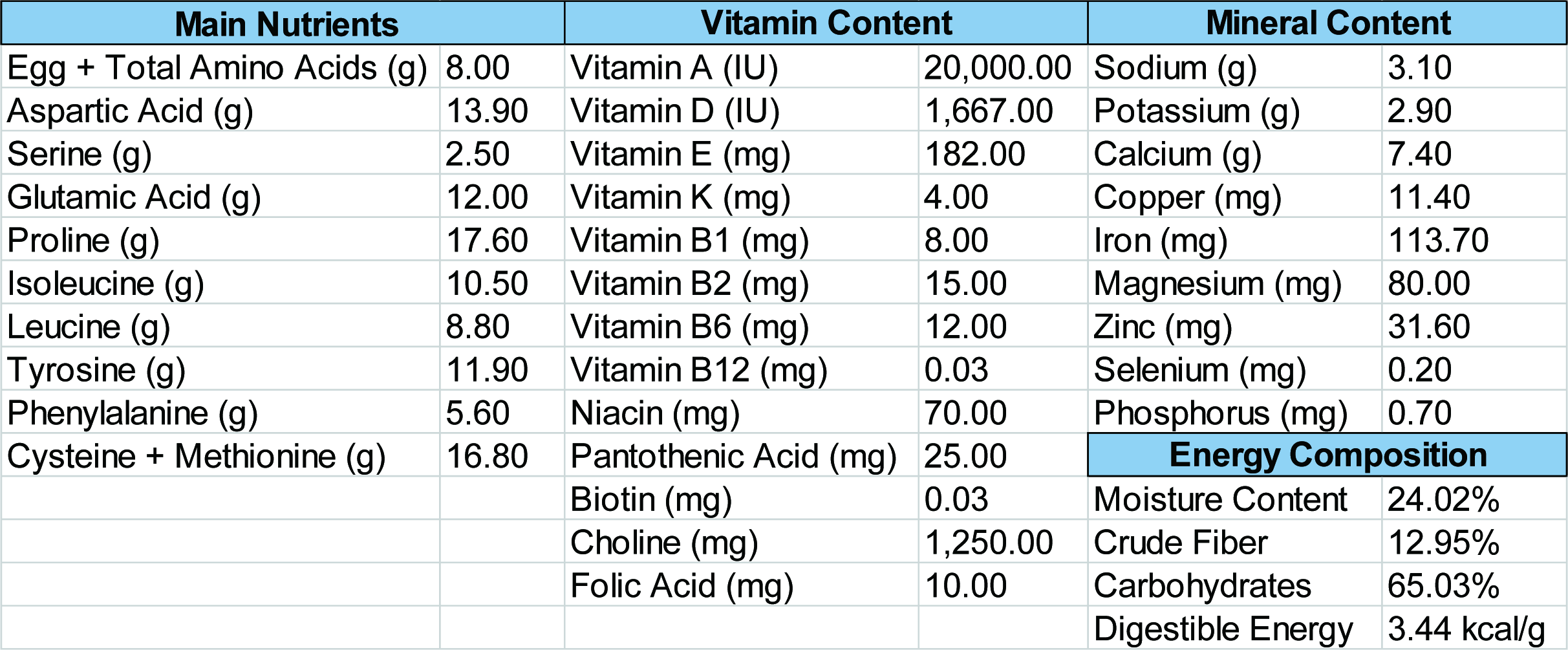

Supplement: Supplementary file 2 — Supplementary Table 1. Detailed nutritional analysis values of mouse diet (per kilogram content) [file 41419_2025_8249_MOESM2_ESM.tif]

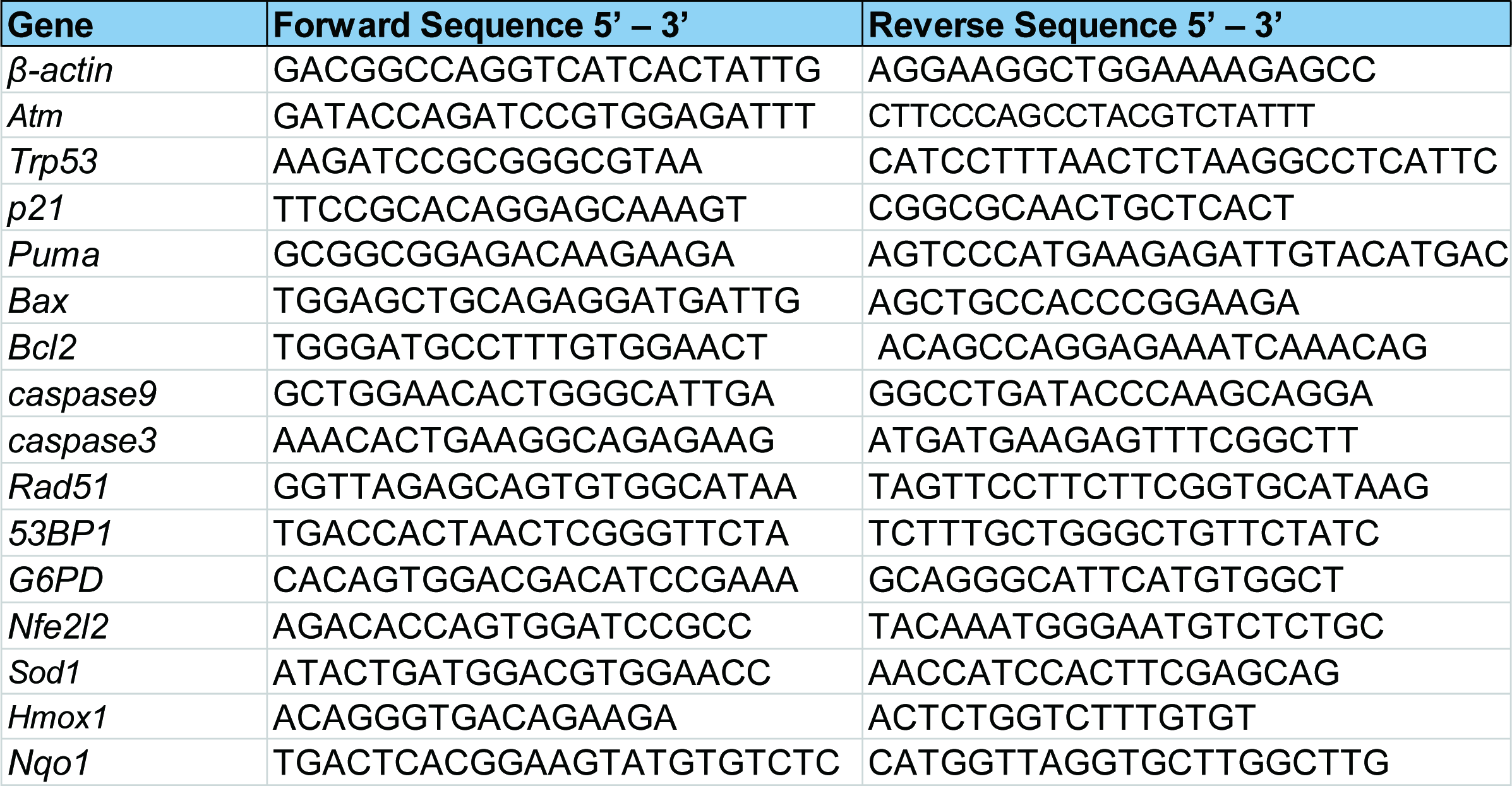

Supplement: Supplementary file 3 — Supplementary Table 2. Primer sequences [file 41419_2025_8249_MOESM3_ESM.tif]

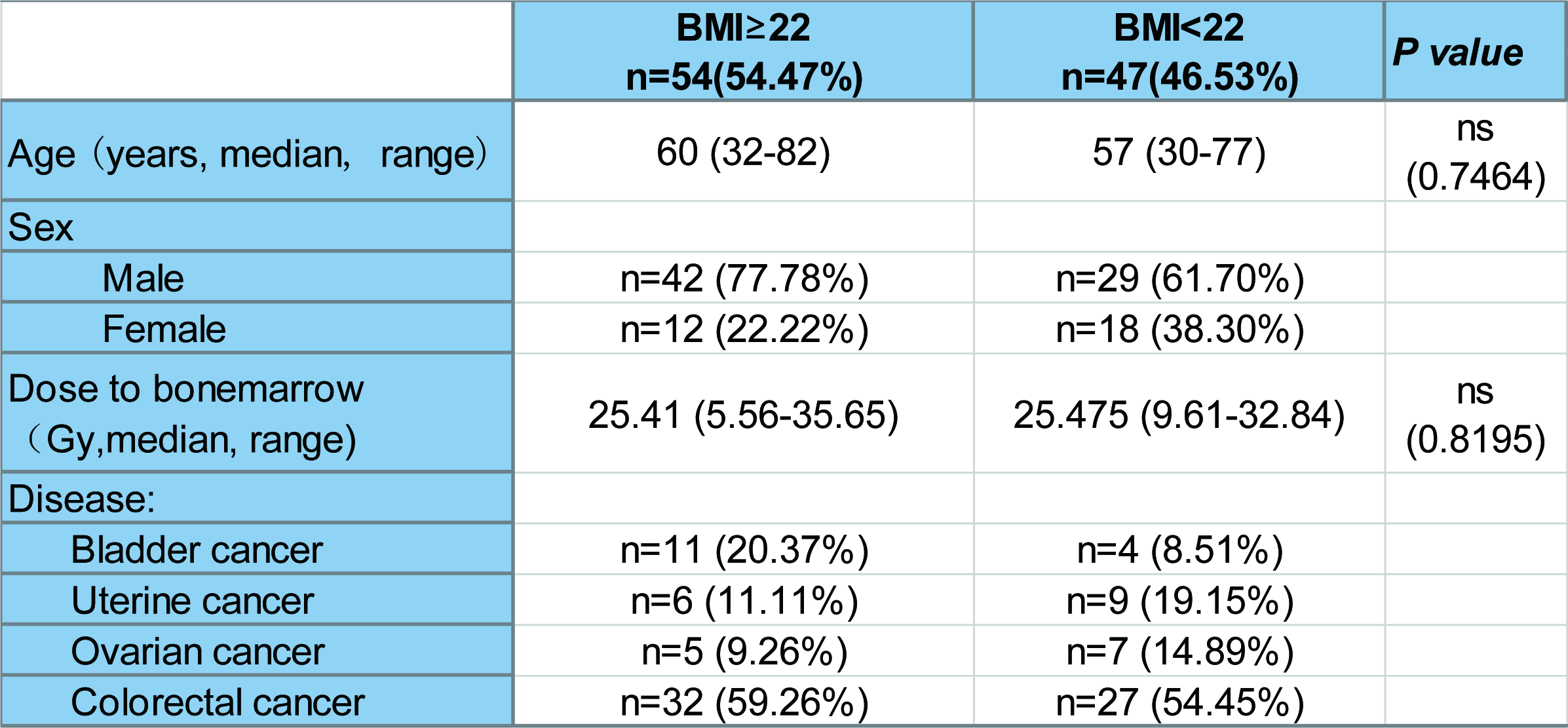

Supplement: Supplementary file 4 — Supplementary Table 3. Baseline demographics, disease characteristics, and irradiation doses to pelvic bone marrow of patients (n = 101) [file 41419_2025_8249_MOESM4_ESM.tif]
